# Parasite worm antigens instruct macrophages to release immunoregulatory extracellular vesicles

**DOI:** 10.1002/jev2.12131

**Published:** 2021-08-16

**Authors:** Amin Zakeri, Bradley J. Whitehead, Allan Stensballe, Clarize de Korne, Andrew R. Williams, Bart Everts, Peter Nejsum

**Affiliations:** ^1^ Department of Clinical Medicine Aarhus University Aarhus Denmark; ^2^ Department of Medicine and Health Technology Aalborg University Aalborg Denmark; ^3^ Department of Parasitology Leiden University Medical Centre Leiden Netherlands; ^4^ Interventional Molecular Imaging laboratory Department of Radiology Leiden University Medical Centre Leiden Netherlands; ^5^ Department of Veterinary and Animal Sciences Faculty of Health and Medical Sciences University of Copenhagen Frederiksberg Denmark

**Keywords:** extracellular vesicles, inflammation, macrophages, reactive oxygen species, *Trichuris suis* soluble products

## Abstract

Emerging evidence suggests that immune cells not only communicate with each other through cytokines, chemokines, and cell surface receptors, but also by releasing small membranous structures known as extracellular vesicles (EVs). EVs carry a variety of different molecules that can be taken up by recipient cells. Parasitic worms are well known for their immunomodulatory properties, but whether they can affect immune responses by altering EV‐driven communication between host immune cells remains unclear. Here we provide evidence that stimulation of bone marrow‐derived macrophages (BMDMs) with soluble products of *Trichuris suis* (TSPs), leads to the release of EVs with anti‐inflammatory properties. Specifically, we found that EVs from TSP‐pulsed BMDMs, but not those from unstimulated BMDMs can suppress TNFα and IL‐6 release in LPS‐stimulated BMDMs and BMDCs. However, no polarization toward M1 or M2 was observed in macrophages exposed to EVs. Moreover, EVs enhanced reactive oxygen species (ROS) production in the exposed BMDMs, which was associated with a deregulated redox homeostasis as revealed by pathway analysis of transcriptomic data. Proteomic analysis identified cytochrome p450 (CYP450) as a potential source of ROS in EVs from TSP‐pulsed BMDMs. Finally, pharmacological inhibition of CYP450 activity could suppress ROS production in those BMDMs. In summary, we find that TSPs can modulate immune responses not only via direct interactions but also indirectly by eliciting the release of EVs from BMDMs that exert anti‐inflammatory effects on recipient cells.

## 1 INTRODUCTION

Macrophages (MΦs) represent a pivotal group of immune cells, which generally can be categorized into pro‐inflammatory (‘M1’) MΦs with potent anti‐bacterial and inflammatory properties or anti‐inflammatory (‘M2’) MΦs, which induce regulatory responses and have wound‐healing properties (Wynn et al., 2013). An imbalance in pro‐ or anti‐inflammatory properties of MΦs is widely appreciated to be an important driver of various diseases including autoimmune diseases and cancer; therefore, there is an urgent need to explore novel ways to resolve MΦs dysregulation (Wynn et al., 2013). Parasitic worms (helminths) are pathogens that profoundly suppress host inflammatory signals and redirect immune responses towards a regulatory phenotype to ensure survival (Zakeri et al., 2018). Moreover, MΦs conditioned by helminth‐derived molecules have been shown to reduce disease severity in several experimental models of inflammatory disorders (Steinfelder et al., 2016). Helminth stimulated dendritic cells (DCs) and MΦs secrete reduced amounts of pro‐inflammatory cytokines (TNFα, IL‐1β, and IL‐6) and have an attenuated capacity to prime pro‐inflammatory T cell responses (Steinfelder et al., 2016). The porcine whipworm *Trichuris suis* shows strong immunomodulatory activity with infection leading to down‐regulation of mucosal inflammatory pathways in pigs (Myhill et al., 2018). Soluble products from *T. suis* (TSPs) not only partially desensitize MΦs in response to inflammatory agonists (preventive effects) but also are able to suppress overactivation of MΦs already exposed to stimulatory agents (our unpublished data). Although most of these studies focused on probing the classical effects of helminth antigens on host immune cells at the protein secretion and gene expression level, it is unknown whether these antigens can affect other aspects of host cell biology including those involved in cell‐to‐cell communication mediated by extracellular vesicles (EVs).

Extracellular Vesicles are heterogeneous membranous compartments that are released by cells into the external environment to mediate intercellular communication. Three main types of EVs have been recognized: exosomes, microvesicles, and apoptotic bodies, that are different in size, surface and intra‐vesicular content, and subcellular origin (Tkach & Théry, 2016). Exosomes originally stem from intraluminal vesicles upon the fusion of multivesicular bodies (MVBs) with the plasma membrane loading components associated with the endocytic network, including foreign captured molecules trafficked to the MVB (Tkach & Théry, 2016), while microvesicles are generated by budding off from the membrane. Prior to 2018 most publications refer to ‘exosomes’, but discriminating exosomes from microvesicles is somewhat controversial due to overlaps in size, composition, and marker proteins (Théry et al., 2018). Thus, we only use the term EVs in accordance with Information for Studies of Extracellular Vesicles (ISEV) criteria (Théry et al., 2018).

Extracellular Vesicles range in size between 50 and 1000 nm diameter and are capable of carrying a wide variety of bioactive compounds, including genetic material (different types of RNAs and DNA), proteins, lipids, and carbohydrates. EVs are released from both hematopoietic and non‐hematopoietic cells into the pericellular environment (Tkach & Théry, 2016). Many studies have shown that EVs actively participate in cellular communication involved in the regulation of immune responses, including antigen presentation, and cellular differentiation (Tkach & Théry, 2016). In terms of immunological effects, EVs can activate or suppress recipient cells by transporting various signalling molecules, including co‐stimulatory and co‐inhibitory molecules. The content of released EVs is modulated by cell activation status and the type of stimuli that donor cells are exposed to (Buzas et al., 2014).

Depending on the context, pathogens can stimulate MΦs to release EVs with stimulatory or regulatory properties. Several studies have also demonstrated that MΦs as recipient cells can be manipulated by EVs released from other cells (Wang et al., 2020). This has driven a surge of interest to explore EV‐mediated MΦ modulation. For example, EVs derived from *Trypanosoma cruzi* trypomastigote‐infected MΦs were found to harbour parasite‐associated antigens and induce inflammatory responses in human MΦs via a Toll‐like receptor 2 (TLR2)‐dependent mechanism (Cronemberger‐Andrade et al., 2020), while the infective stage of this protozoan parasite induces the release of immunomodulatory EVs coated with TGF‐β from MΦs and lymphocytes to promote invasion (Cestari et al., 2012; Gavinho et al., 2018). Likewise, *Salmonella typhimurium‐*infected MΦs were found to secrete pro‐inflammatory EVs inducing TNFα release via a TLR4‐dependent pathway (Hui et al., 2018).

Similarly, *Mycobacterium tuberculosis*‐infected MΦs shed EVs containing mycobacterial antigens that not only induce a pro‐inflammatory response in naive MΦs in a MyD88‐dependent manner, but also activate antigen‐specific T cells. MΦs infected with *M. tuberculosis* secrete EVs that suppress the expression of major histocompatibility complex (MHC) class II molecules in BMDMs through TLR2‐driven signalling (Wang et al., 2019). Helminths have also been shown to induce the release of regulatory EVs from host DCs. Treatment of bone marrow‐derived DCs (BMDCs) with *Schistosoma japonicum* soluble egg antigen enables them to produce EVs capable of attenuating dextran sulphate sodium‐induced colitis in mice (Wang et al., 2017).

Although the suppressive effects of TSPs on MΦs and DCs and potential mechanistic pathways have been explored (Hoeksema et al., 2016; Laan et al., 2017), it is unknown whether the nature and functional properties of EVs released by MΦs can be modulated by TSP exposure. To address this question, we explored the effects of small EVs (sEVs) and large EVs (lEVs) shed by TSP‐pulsed MΦs on LPS‐induced inflammatory responses in naive BMDMs and BMDCs. Our findings demonstrate that sEVs and lEVs derived from TSP‐pulsed BMDMs suppress inflammatory cytokines (TNFα and IL‐6) and surface markers (CD86 and MHCII) in inflammatory BMDMs and BMDCs and promote ROS accumulation in these cells relative to EVs derived from unstimulated MΦs. In contrast, no effect on other markers, including YM‐1, CD206, RELMα, and PDL‐2, was observed, indicating a lack of M2 phenotype polarization. Comparative proteomic analysis of sEVs from naive, LPS‐, and TSP‐pulsed MΦs, revealed the presence of a reactive oxygen species (ROS) inducer enzyme, CYP450, in sEVs from TSP‐pulsed MΦs, which might be responsible for ROS induction and glutathione pathway suppression in naive MΦs or vice versa. Interestingly, blocking this enzyme with a specific inhibitor, 1‐Aminobenzotriazole (1‐ABT), reduced ROS in recipient cells after exposure to sEV from TSP‐pulsed MΦs. Together, our findings suggest that modulation of host EVs presents a novel mechanism by which helminths subvert host immune responses.

## 2 MATERIALS AND METHODS

### 2.1 Preparation of helminth soluble product

TSPs were prepared as previously described by Kuijk *et al*. (Kuijk et al., 2012). Briefly, adult *T. suis* were collected from the caecum and proximal colon of infected pigs and washed thoroughly with saline three times and then eight times with RPMI 1640 medium supplemented with antibiotics over a period of 4 h, all at 37°C, 5% CO_2_. TSPs were prepared by homogenizing whole worms in phosphate‐buffered saline (PBS) as reported by Kuijk *et al*. (Kuijk et al., 2012). Protein concentration was measured using bicinchoninic acid assay (BCA) (Thermo Fisher) and endotoxin level was evaluated by LAL assay (Thermo Fisher), which was below detection limits.

### 2.2 BMDMs, BMDCs, and RAW264.7 macrophages cell culture

RAW264.7 macrophages (ATCC, Manassas, VA, USA) were cultured in Dulbecco's Modified Eagle Medium (DMEM) (GIBCO BRL, Life Technologies, Inc.) containing 10% fetal bovine serum (FBS) (GIBCO BRL, Life Technologies, Inc.) and penicillin/streptomycin (100 U/ml: 100 U/ml; Sigma–Aldrich) at 37°C in 5% CO_2_ incubator.

Murine bone‐marrow‐derived macrophages (BMDMs) and dendritic cells (BMDCs) were obtained as described previously (Weischenfeldt & Porse, 2008). Briefly, to generate BMDMs and BMDCs, 8‐week‐old C57BL/6 mice were sacrificed, then femurs and tibias were separated and washed with PBS and disinfected with 70 % ethanol. Subsequently, bone marrow cells were harvested in a sterile 50 ml conical centrifuge tube on ice. Cells were centrifuged for 5 min at 300 × g and after removing the supernatant, the cells were resuspended in RBC lysis buffer for 1 min at room temperature. After that, RPMI 1640 (Gibco/Life Technologies) containing 10% FBS and 100 g/ml penicillin‐streptomycin (Life Technologies) was added and centrifuged for 5 min at 300 g. The supernatant was removed, and the cell pellet was resuspended in RPMI 1640 and transferred to sterile Petri dishes. To generate BMDMs and BMDCs, the BM cells were cultured in RPMI 1640 containing 10% FBS supplemented with 20% L929 cell supernatant (containing macrophage colony‐stimulating factor) for BMDMs and 20 ng/ml granulocyte‐macrophage colony‐stimulating factor for BMDCs. The BM cells were cultured in a humidified incubator with 5% CO_2_ at 37°C for 7 days with media exchange every 2 days. The purity of BMDMs and BMDCs was evaluated using two surface markers with fluorochrome‐conjugated anti‐mouse antibodies, including labelled mouse CD11b & F4/80 and CD11c & MHC‐II, respectively. More than 95% of the differentiated cells were positive for corresponded markers.

### 2.3 Small extracellular vesicles (sEVs) and large extracellular vesicles (lEVs) isolation

Confluent monolayers of RAW 264.7 mouse macrophage cell line (60 million cells) were treated with *T. suis* soluble products (TSPs) (20 μg/ml), LPS (10 ng/ml), or left untreated for 4 h in 175 cm^2^ culture flasks, subsequently cells were thoroughly washed three times with PBS, fresh DMEM supplemented with 5% EV‐depleted FBS (Thermofisher, A2720803) was added and the cells were incubated at 37°C in 5% CO_2_ incubator, then the supernatants were collected after 24 h for EV isolation. BMDMs were also plated ∼1.5 × 10^7^ cells in 100 mm Petri dishes. After 4 h at 37°C and 5% CO_2_, the culture medium was discarded and cells were washed three times with PBS (Singh et al., 2011; Singh et al., 2012); then the medium was replaced with RPMI 1640 containing 5% EV‐depleted FBS (Thermofisher, A2720803). The EVs in the medium were isolated using differential ultracentrifugation or size exclusion chromatography (SEC). The EV containing media was sequentially centrifuged at 500 × g for 5 min to eliminate floating cells, 2k × g for 20 min to remove cell debris, and 10k × g for 30 min at 4°C to pellet lEVs. The lEVs were resuspended in particle‐depleted cold PBS, pooled, and concentrated with 100 kDa cut‐off Amicon filters (Merck).

The supernatants of the 10K × g fraction were pooled and concentrated using 100 kDa molecular weight cut‐off Amicon filters; the concentrated sample was subsequently centrifuged in a Beckman Coulter with a TI‐50 rotor (Brea, CA, USA) at 100k × *g*, for 80 min at 4°C to pellet sEVs. Next, the sEVs pellet was washed with filtered (0.2 μm) cold PBS followed by ultracentrifugation, as described previously. Finally, the pelleted sEVs were carefully reconstituted in sterile cold PBS 100 μl or lysed in radioimmunoprecipitation assay (RIPA) buffer, and protein content was measured by the Micro BCA protein assay kit (Thermo Fisher Scientific, MA, USA).

Small extracellular vesicles were also isolated using SEC method with commercially available qEV SEC columns (iZON Science Ltd.) following the manufacturer's protocol. Culture media was subjected to pre‐clearing steps, including centrifugation at 500 × g for 5 min, 2k × g for 20 min, and 10k × g for 30 min at 4°C. The culture supernatants of the 10K × g fraction were pooled and concentrated using 100 kDa cut‐off Amicon filter to a final volume of 500ul; subsequently, the concentrated sample was applied to the SEC column and eluted by adding sterile‐filtered PBS. The sEVs were collected in fractions 7–9 (1.5 ml) and kept at ‐80°C until use.

### 2.4 ROS measurement

ROS measurement was carried out using 2′,7′‐dichlorofluorescein diacetate‐based DCFDA/H2DCFDA‐Cellular ROS Assay (Invitrogen) according to the manufacturer's instructions. DCFDA is passively absorbed by cells and cleaved by intracellular esterases and oxidized by intracellular ROS, generating a highly fluorescent compound, DCF (2′,7′‐dichlorofluorescein). Briefly, 100,000 BMDMs were plated in 96‐well flat‐bottom microplates in RPMI 1640 supplemented with EV‐depleted FBS. BMDMs were pre‐treated with N‐acetyl‐L‐cysteine (NAC) (as an inhibitor of ROS generation) or PBS for 30 min before stimulation with sEVs and LPS for 6 h. BMDMs supernatants were collected for ELISA and media was replaced with 100 μl fresh RPMI 1640 containing DCFDA. BMDMs were treated with 4 μM DCFDA probe and incubated in the dark for 40 min (37°C, 5% CO_2_). Next, BMDMs were centrifuged for 4 min at 1200 rpm and supernatant was discarded. The cell pellet was resuspended in FACS buffer and the DCF level was detected in the green channel (FITC spectrum) using BD LSRII Flow Cytometer (BD, Biosciences).

### 2.5 Nanoparticle tracking analysis

Nanoparticle tracking analysis using a NS300 (NTA) (Malvern, UK) with a 488 nm laser and NTA 3.2.16 software (Malvern) was employed to determine the concentration and particle size distribution of the isolated EVs according to the manufacturer's instructions. EV samples were diluted in particle‐free filtered PBS and 1 ml was loaded into the sample chamber via syringe pump with a constant injection flow rate before video recordings. Each sample analysis consists of five 60 s video captures using camera level 14 and detection threshold of four. Videos were analysed using the following operating script: temperature 25, capture 60, delay 10, and repeat five. The reported results are the average of five 1‐min reads.

### 2.6 Cryo‐EM imaging and transmission electron microscopy

EVs were applied to a glow‐discharged (2 min in 0.2 mbar air using a EMITECH K950X with glow discharger unit) 300 mesh EM grid (Quantifoil R2/2) and were vitrified using an EMGP (Leica, Vienna) at room temperature and 100% humidity. Excess sample was removed by blotting for 1 s with filter paper (Whatman #1). The blotted grids were plunged into liquid ethane (‐182°C). After vitrification, the grid was stored in liquid nitrogen until further use. Grids were mounted in a Gatan 626 cryo‐holder for cryo‐EM imaging. Cryo‐EM imaging was performed on a Tecnai 12 electron microscope (FEI Company, The Netherlands) operated at 120 kV. Images were recorded on a 4k×4k Eagle camera (FEI Company, The Netherlands). Images were recorded at 18,000× magnification (pixel size 1.2 nm) between 5 and 10 μm under focus. Transmission electron microscopy was performed using purified EVs suspended in PBS dropped onto a copper grid coated with formvar and carbon, which was previously glow discharged in air. EVs attached to the grid were rinsed with water and embedded in 2% methylcellulose in water containing 0.6% uranyl acetate and subsequently air‐dried. The EVs were visualized in a Tecnai 12 transmission electron microscope (Thermofisher) operating at 120 kV at 30,000× magnification.

### 2.7 Western blotting

Cell layers or sEV pellets were resuspended in RIPA buffer supplemented with Halt Protease Inhibitor Cocktail (Thermo Fisher); then protein concentration was quantified by Micro BCA Assay kit (Thermo Fisher Scientific, MA, USA). Next, an equal protein amount of each sample was heated (10 min, 70°C) with 4× Laemmli buffer (Bio‐Rad) and DTT, then left at room temperature for 5 min before loading on 12% sodium dodecyl sulphate‐polyacrylamide gel electrophoresis (SDS‐PAGE). Subsequently, the proteins were transferred onto polyvinylidene difluoride (PVDF) membranes and blocked with 5% BSA in 1× TBST for 1h at room temperature. Membranes were probed with primary antibodies; HSP90, CD63, ALIX, Calnexin, Calreticulin, overnight at 4°C with rocking. Antibody dilutions were prepared in 1× TBST with 1% BSA. Next day, TBS‐T buffer was used to wash membranes three times and then incubated with horseradish peroxidase (HRP)‐conjugated goat anti‐rabbit secondary antibody (Sigma Aldrich, Germany) at room temperature. All primary antibodies, except HSP90 (Santa Cruz, sc‐7947), were purchased from Cloud clone corporation (CCC, USA) and diluted at 1:1,000 and secondary antibody used at a 1:2000 dilution. After 1 h, membranes were washed three times with TBST buffer, the protein signals were detected after exposure to enhanced chemiluminescence reagents (Cell Signalling Technology, Boston, USA) using the ChemiDoc system (BioRad).

### 2.8 sEVs labelling

BMDM and RAW‐derived sEVs (∼10^8^ particle/ml or concentration 5 μg/ml) were labelled using the PKH26 kit (Sigma‐Aldrich) after the first ultracentrifugation step at 100k × *g*. Briefly, sEV pellet from the first ultracentrifugation step was resuspended in 80 μl of Diluent C, then 93 μl of mixed PKH26/Diluent C (3 μl in 200 μl Diluent C) was added and incubated 3 min at RT in the dark. The same procedure was carried out for the ‘dye control’ where the supernatant of the first ultracentrifugation step was used. Next, 7 ml of cold filtered PBS was added to stop the labelling process and followed by ultracentrifugation at 100k × *g* for 80 min. The labelled sEV pellet and dye control were resuspended in PBS and used for *in vitro* uptake experiments. Labelled sEVs were added to the culture medium of BMDMs and BMDCs (5 × 10^4^ cell/well) with a concentration of 2 × 10^3^ particles per recipient cell.

### 2.9 Cell stimulation

BMDMs and RAW 264.7 macrophages were seeded into 48‐well culture plates (Sarstedt, Germany) at a density of 100,000 cells/well. Cells were incubated for 2 h at 37 °C and 5% CO_2_ to allow attachment. Thirty minutes before stimulation with LPS 10 ng/ml, cells were treated with the given number of EVs derived from TSP‐pulsed, LPS‐pulsed or untreated MΦs. After 24 h, the supernatants were collected for cytokine measurement and cells were detached for flow cytometry.

### 2.10 Flow cytometry on sEVs

After ultracentrifugation RAW cell‐derived EVs were resuspended in 100 μl of filtered PBS. Conjugated antibodies including 1ul mouse anti‐CD9 antibody (AF647; BioLegend, cat. 124809) or isotype control (Mouse IgG2a, κ isotype Ctrl) were added. The mixture of sEVs and antibodies was incubated at room temperature for 20 min, then 1 ml filtered PBS was added and centrifuged at 16,000 × g for 20 min to exclude free antibodies. Controls included 0.1% Triton X‐100‐treated sEV (permeabilization buffer), sEVs alone, antibodies alone following the described procedure (Gray et al., 2015). After centrifugation, 1 ml of supernatant was discarded and 100 μl of filtered PBS was added and resuspended before detection with Cytek Aurora flow cytometry. Single labelled and non‐labelled compensation bead controls were also used for optimization.

### 2.11 Flow cytometry on BMDMs and BMDCs

To block Fc binding and to discriminate dead cells, BMDMs were incubated with anti‐mouse FcR antibody (eBioscience) and Aqua live/dead staining (Invitrogen) for 30 min at 4°C in FACS buffer; subsequently, cells were washed three times with FACS buffer. Next, cells were washed and fixed with 2% paraformaldehyde for 10 min at room temperature prior to intracellular staining. Cells were permeabilized with 1X eBioscience Permeabilization buffer for 5 min at room temperature. Lastly, cells were stained in a final volume of 50 μl FACS buffer containing labelled antibodies in two different panels for 30 min at 4°C. The list of antibodies used for staining includes: anti‐CD11b‐PE‐Cy7 (eBio, Cat# 25‐0112‐82); anti‐F4/80‐APC‐Cy7 (eBio, Cat# 47‐4801‐82); anti‐MHCII‐APC (eBio, Cat# 17‐5321‐82); anti‐CD86‐PE (BD Biosciences, Cat# 553692); anti‐PDL‐2‐Percp‐Cy5.5 (eBio, Cat# 46‐9972‐80); anti‐NOS2‐PE (eBio Cat# 12‐5920‐80), anti‐YM‐1‐Percp‐Cy5.5 (Biolegend, cat# 405214); anti‐RELMα‐APC (Invitrogen, Cat# A21244); anti‐CD206‐FITC (Biolegen, Cat# 141703). To set up the machine, unstained cells and single fluorochrome stained BDCompBeads Compensation Particles (BD Biosciences) were included. The BD FACSCanto II or BD LSRII Flow Cytometer (BD, Biosciences) was used, and results were analyzed with FlowJo software version 7.6.5 (Tree Star). Results are reported as geometric mean fluorescence intensity (MFI). Fluorescence‐minus‐one (FMO) controls were used for gating positive cells. BMDMs were identified as F4/80^+^ CD11b^+^ cells.

### 2.12 Microarray gene expression

RAW 264.7 macrophages were seeded at 2 × 10^5^ cell/well in a 48‐well plate with culture media (DMEM + 5% EV‐depleted FBS) and stimulated with 24,000 EVs/recipient cell for 6 h. Three types of EVs were used for stimulation, including TSP‐sEVs, UNS‐sEVs, and LPS‐sEVs in the presence or absence of LPS. RAW 264.7 macrophages were collected after 6 h and total RNA was isolated using Mini RNeasy columns (Qiagen). RNA concentration and quality were analysed by the RNA 6000 Nano Chip (Agilent), and only samples with an RNA Integrity Number (RIN) > 7 were used. RNA was processed and hybridized to the Affymetrix Mouse chips at the Eurofins Genomics Microarray facility, Aarhus, Denmark. Student's *t*‐test was used to evaluate differences in individual gene expression values between the groups. Significantly regulated genes were defined as ±2‐fold change in mean expression compared to untreated cells and a *P*‐value < 0.05. Gene ontology (GO) and Kyoto Encyclopedia of Genes and Genomes (KEGG) analysis was performed using the Gene Set Analysis Toolkit (GSEA) software. For this analysis, entire genes were selected for analysis. Detected KEGG pathways were ranked in order of adjusted *p*‐values, referring to pathway impact analysis or probability of a pathway being significantly regulated.

### 2.13 ELISA

The concentration of TNFα, IL‐10, and IL‐6 released by MΦs was measured by ELISA (R&D Systems, Minneapolis, MI, USA), as per the manufacturer's protocol and the concentrations were determined against a standard curve.

### 2.14 Proteomics analysis of sEVs by LC‐MS/MS

Equal amount of each sample (30 μl) was lysed in 0.1% ProteaseMax in 0.1 M TEAB. Then, samples were sonicated for 3 min in water heated for 10 min to denaturize and stored at ‐80 for further analysing. Using a nanodrop 1000 UV‐vis spectrophotometer (Thermo Scientific, Waltham, MA, USA) protein concentration was measured. To reduce the samples, they were kept at 37°C for 30 min with 10 mM tris (2‐carboxyethyl) phosphine (Thermo Scientific, Waltham, MA, USA) and 50 mM chloroacetamide (Sigma Aldrich, St. Louis, MO, USA). Samples were then subjected to digestion with 1 μg of sequencing grade modified trypsin in 0.1 M TEAB (Promega, Madison, WI, USA) and acidified with 0.1% trifluoroacetic acid and reduced by vacuum centrifugation. To dissolve the reduced sample, 30 μl 2% acetonitrile; 0.1% formic acid; 0.1% trifluoroacetic acid was used then sonicated in the water bath for 5 min. Dionex RSLC UPLC system (Thermo Scientific, Waltham, MA, USA) with uPAC 50 cm analytical column with precolumn (Pharmafluidics, Ghent, Belgium) were used to separate the samples. Then, samples were run based on the following settings 5 min at 10 μl per min and the mobile phase was ramped over 30 min at a constant flow rate of 700 nl/min from 98% solvent A (0.1% formic acid) and 2% solvent B (0.1% formic acid in acetonitrile) to 45% solvent B in 40 min. Lastly, the peptides which were already eluted directly ran in coupled ThermoSci QE HF‐X mass spectrometer (Thermo Scientific, Bremen, Germany). These settings were selected in positive mode based on data‐dependent acquisition method: MS2‐scan resolution 30,000; MS1‐scan resolution 120,000; isolation window m/z 1.6; NCE 28, and mass range m/z 375–1200. Peptide hits were searched against *Mus musculus* UniProt protein entries (released 05/2019) using standard settings in Maxquant v.1.6.12.0. Proteins of interest were analysed using STRING and GO.

### 2.15 Bioinformatic analyses of the proteomic data

Protein‐protein interaction networks of the identified proteins were created using STRING database with default parameters and visualized using Cytoscape software 2.8. Data were filtered based on hits with a minimum of three peptides and a score of 20. Using GSEA, GO annotations were applied to analyse all identified proteins and probe both molecular functions and cellular components of EV proteins. KEGG analysis was used to identify the potential signalling pathways. A mouse genome was uploaded as a reference gene list (STRING v.11) for identifying enriched gene sets. The top GO terms were chosen based on their statistical significance (*P* < 0.05). Venn diagrams of the results were plotted by using Venny software (version 2.1).

### 2.16 Seahorse extracellular flux analysis

BMDMs were plated at 7 × 10^4^ cells/well into XFe96 cell culture microplates (Agilent) and were allowed to adhere during 4h incubation at 37 °C and 5% CO_2_. Next, BMDMs were stimulated with PBS and sEVs from TSP‐pulsed or untreated BMDMs for 24h. Sensor cartridge (Agilent Technology) was pre‐incubated the day before running the XF Assay with 200 μl/well XF96 calibrant solution on one utility plate overnight at 37 °C in a non‐CO_2_ humidified incubator. Cell culture media was replaced with RPMI 1640 (GIBCO) supplemented with 1% L‐glutamine + 5% FCS and incubated for 1h at 37 °C without CO2. To measure oxygen consumption rate (OCR) and extracellular acidification rate (ECAR), different components were prepared, including 10 mM glucose, 1μM Oligomycin, 2μM Carbonyl cyanide‐p‐trifluoromethoxyphenylhydrazone (FCCP), and 1μM Rotenone/Antimycin A (R/AA) with the Seahorse XFe‐96 Bioanalyzer (Agilent) and were analysed using XFe Wave software. Each measurement cycle consisted of 3 min mix, 0 min wait, and 3 min measure. After the measurement, cell numbers were counted and all the results were normalized to the number of cells.

### 2.17 sEVs uptake by macrophages

BMDMs, BMDCs, and RAW cells (5 × 10^4^cell/well) were seeded in a 96‐well optical‐bottom plate (Thermo Fisher Scientific) and incubated overnight. The cells were incubated for 2h at 37°C with PKH26‐labelled sEVs from TSP‐treated, LPS‐treated, and untreated MΦs and subsequently washed. Latrunculin A (5uM) was used to inhibit the vesicle uptake and was added to the cells 30 min before adding sEVs. The EV‐depleted fraction was included as dye control. The cells were counterstained with Hoechst (1:100, stock: 1 mg/ml, Sigma‐Aldrich) before imaging. Images were taken at 37°C on a Leica TCS (true confocal scanning) SP8 WLL (white light laser) microscope (Leica Microsystems). The sequential scanning mode was applied to image Hoechst (excitation: 405 nm, emission: 420–470 nm) and PKH26 (excitation: 561 nm, emission: 570–620 nm). For imaging, the uptake of EVs, a 63x objective (Leica HC PL APO 63x/1.40na OIL CS2) was used. Z‐stacks were recorded and 3D images were generated using the Leica software (LAS X version 1.1.0.12420; Leica Microsystems).

### 2.18 Statistical analysis

Student's *t‐*test was used to compare two groups and one‐way ANOVA was used for more than two groups. Bonferroni correction was used to adjust for multiple testing. Data were analysed with Graphpad version 9 software, and statistical *P‐*values of *<* 0.05 were considered significant. Each experiment was conducted two or three times and represented as mean ± SD.

## 3 RESULTS

### 3.1 Isolation and characterization of sEVs and lEVs from TSP‐pulsed BMDM and RAW cells

**FIGURE 1:**
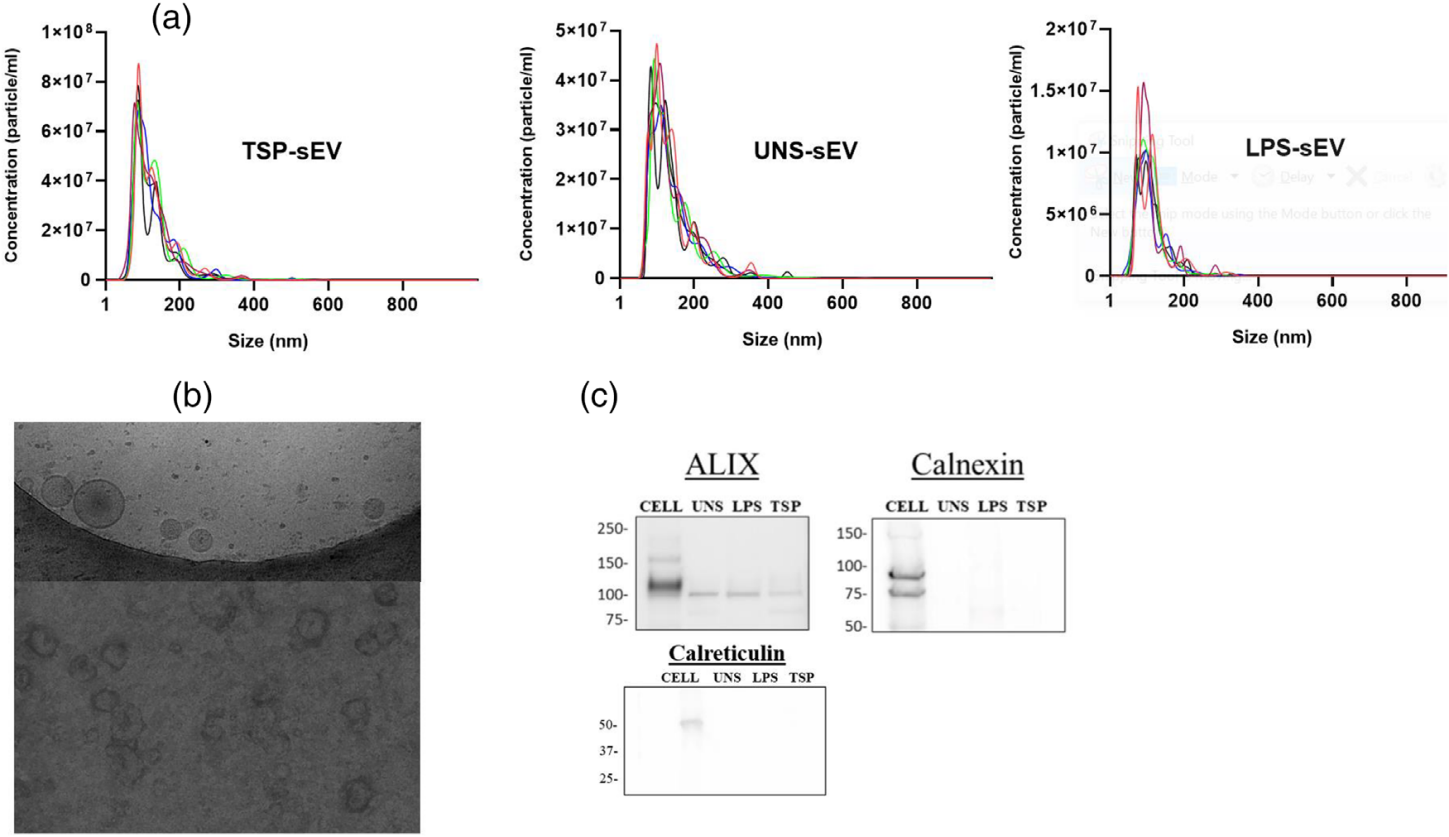
Isolated sEVs show the main features of small vesicles. (a) NTA data showing the size and concentration distribution of sEV derived from BMDMs pulsed with TSP, LPS, or without stimulation after a 24 h period of culture. (b) Cryo‐EM and TEM micrograph of sEVs from BMDM cells. (c) Western blot analysis of sEV markers, Alix, Calnexin, and Calreticulin in BMDM‐sEV and cell lysate (representative of two independent experiments)

We isolated sEVs from supernatants of cultured mouse BMDMs and RAW 264.7 macrophages with and without TSP stimulation. TSPs did not induce cytotoxicity or cell death (Figure S1A). sEVs generated by TSP‐treated and naive MΦs cultures were purified using ultracentrifugation. SEVs derived from BMDMs isolated by ultracentrifugation were analysed using NTA to quantify EVs (particle number) and compare their size differences. Particle concentration was found to be 4.93 × 10^9^, 7.39 × 10^8^, and 3.42 × 10^9^ per millilitre and average modal size of 85.4 ± 2.1 nm, 98.4 ± 4.9 nm, and 84.5 ± 5.1 nm for TSP‐ (TSP‐sEVs), LPS‐stimulated (LPS‐sEVs), and unstimulated BMDMs (UNS‐sEVs), respectively (Figure 1a). These results are consistent with the 30–100 nm size range expected for these types of EVs (Bachurski et al., 2019).

TEM micrographs of TSP‐sEVs and UNS‐sEVs along with cryo‐EM confirmed the presence of round‐shaped vesicles within the size range of sEVs (50–150 nm) (Figure 1b). Furthermore, to validate the purity of sEVs, Western blotting was applied to check for the presence of canonical sEV proteins, including alix, calreticulin, and the lack of cell‐associated marker calnexin (Figure 1c). The observation that the sEVs harboured both surface and luminal markers such as HSP90 and CD63 but no calnexin confirmed the purity of the sEVs (Figure S1B). Flow cytometry and quantitative proteomic analyses (see below) confirmed the presence of tetraspanin CD9 and other canonical sEV markers in the samples (Figure S1C). Thus, the identified morphology based on TEM (Figure S1D) and NTA along with the presence of sEV protein markers supports successful isolation of sEVs. In addition, lEVs were isolated by differential centrifugation and showed a median size of 100–200 nm based on NTA (Figure S1E).

### 3.2 TSPs increase the release of sEVs from macrophages

It has already been shown that some pathogens, such as the protozoan *T. cruzi*, increase the release of EV from host MΦs (Cronemberger‐Andrade et al., 2020). To investigate whether pulsing MΦs with TSPs may affect the quantity or quality of the released sEVs, BMDMs were either treated with TSPs, LPS, or left untreated for 4 h. sEVs were isolated from the supernatants of MΦs via differential ultracentrifugation and quantified using NTA. Our data showed a size distribution of 50–200 nm for all sEVs (Figure S2A). The number of sEVs released by TSP‐pulsed MΦs was three times higher than sEVs generated by LPS‐stimulated or unstimulated MΦs (Figure S2B). In addition, to evaluate whether this effect is influenced by the isolation method, we isolated sEVs using SEC and observed the same proportions of isolated sEVs (data not shown).

### 3.3 Pathway modelling and functional interpretation of sEV proteins

EVs play an important role in trafficking host and pathogen‐derived proteins between immune cells. To further characterize the content of the sEVs released by TSP‐treated BMDMs a liquid chromatography‐tandem mass spectrometry (LC‐MS/MS) based proteomic analysis was performed. A total of 693, 102, and 108 unique proteins were identified in TSP‐, UNS‐, and LPS‐sEV, respectively (Figure 2a). sEVs released by TSP‐pulsed macrophages had significantly more unique proteins than sEVs from unstimulated macrophages. Four‐hundred and five proteins were detected as shared proteins between all sEVs.

FIGURE 2Shared and unique proteins identified in sEVs. (a) Proteomics analysis of sEVs isolated from RAW 264.7 cell cultures stimulated with TSP, LPS or unstimulated for 24h. Liquid chromatography–tandem mass spectrometry (LC‐MS/MS) analysis was used to identify the sEV proteins. Venn diagram indicates the shared and unique proteins identified in sEVs derived from LPS‐, TSP‐stimulated and unstimulated macrophages. TSP could induce the highest number of unique proteins relative to LPS and unstimulated. (b) GO terms of shared sEV proteins identified by proteomics. Their GO terms including biological process, molecular function, and cellular components were analysed. The top GO terms were chosen in terms of the statistical significance (the smallest P‐value), and the fold enrichment for each term is shown. (c) the protein‐protein interaction network of shared proteins sorted into sEV was generated by STRING database using default parameters. The main category of networks was extracted which proteins involved in endocytosis vesicle, extracellular organelle, and polysome

**Figure:**
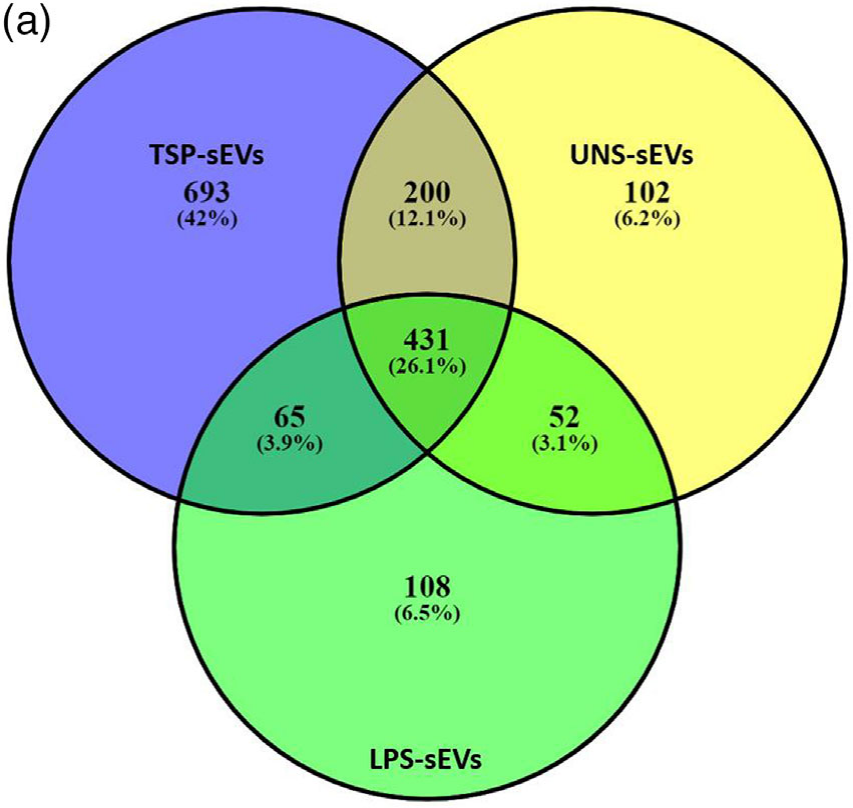

**Figure:**
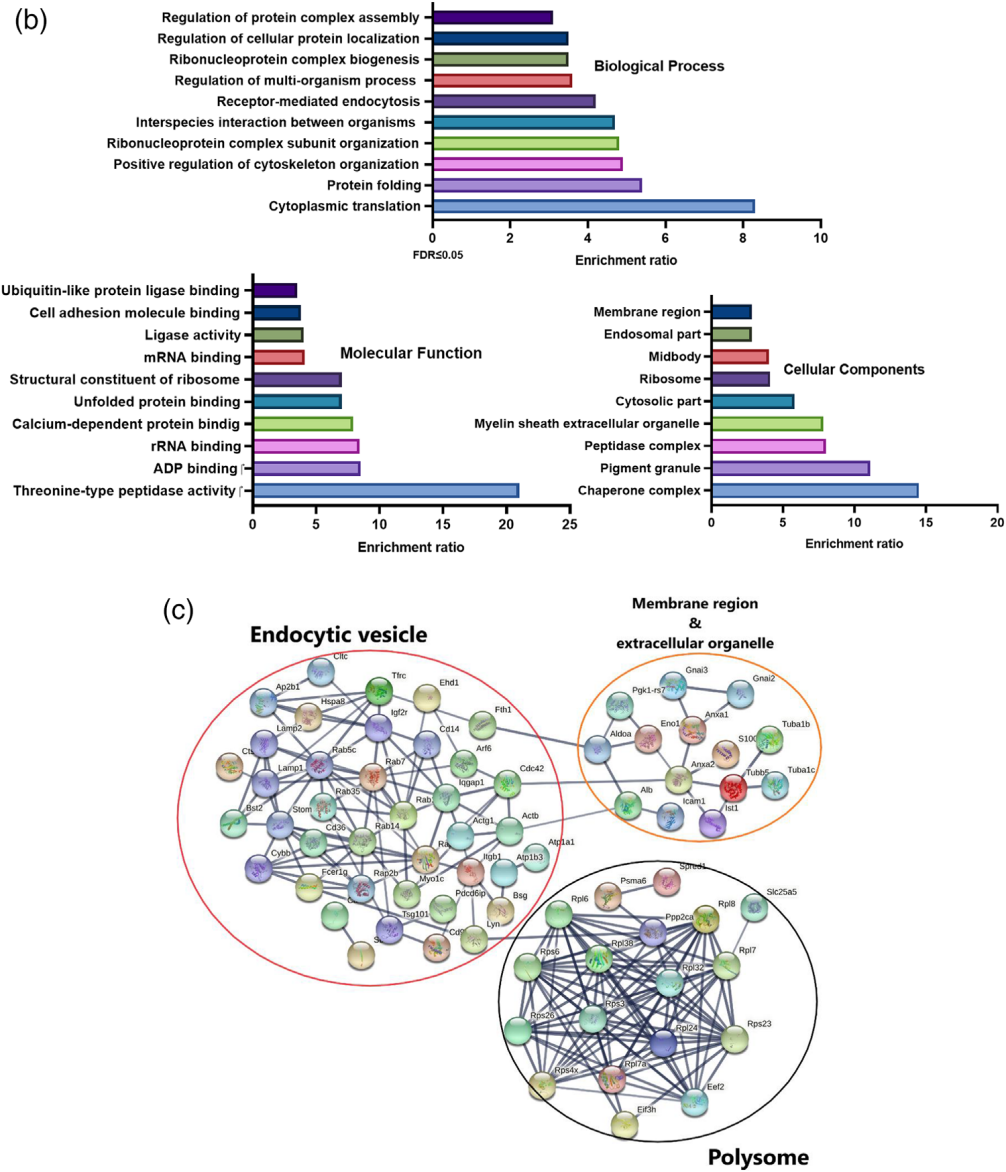

**FIGURE 3:**
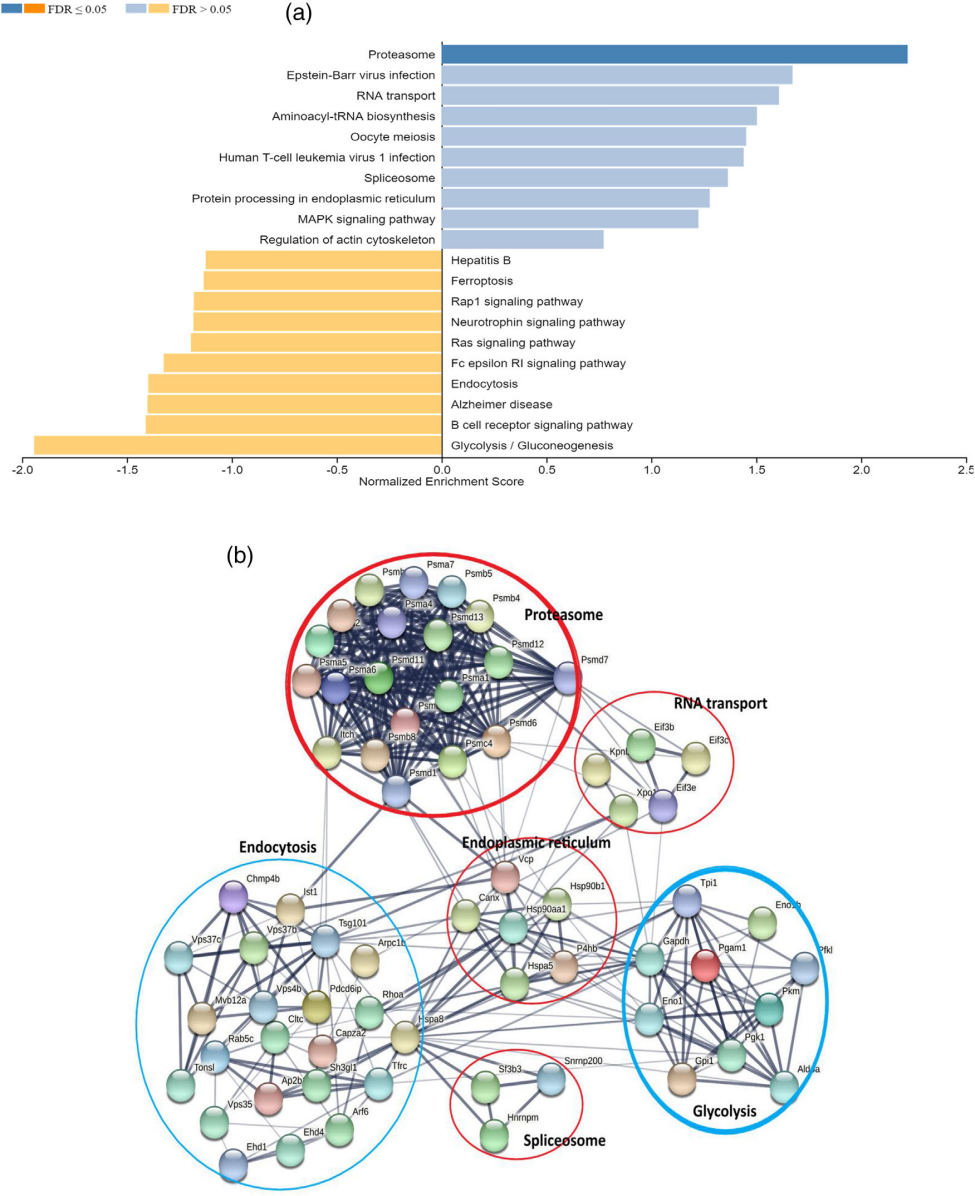
Analysis of protein enrichment in TSP‐sEV versus UNS‐sEV. (a) The relative protein level was quantified by LC‐MS analysis. Proteins enriched in TSP‐sEV compared with UNS‐sEV were analysed using Reactome pathway modelling. Then, proteins involved in top enriched signallings such as endocytosis, proteasome, and endoplasmic reticulum were selected for network analysis. The top upregulated pathway was proteasome and downregulated signalling was glycolysis in TSP versus UNS‐sEV (FDR ≤ 0.05). (b) The protein‐protein interaction network of up‐ and downregulated proteins in sEVs was analysed by STRING database using default parameters. As illustrated downregulated networks were indicated with blue circle while upregulated pathways were separated with red circle

Gene Ontology analysis of the proteome of TSP‐sEVs and UNS‐sEVs revealed the presence of proteins involved in the biological process including cytoplasmic translation, endocytosis, and biogenesis (Figure 2b). Using GO we annotated proteins that are part of the cellular components including chaperon complex, extracellular organelle, and endosomal part. Moreover, we identified proteins that are associated with molecular functions (Figure 2b) including rRNA binding, ADP binding, peptidase activity, unfolded proteins, mRNA binding, and cell adhesion molecular binding (Figure 2b). Potential multiple interactions among proteins related to sEVs were also mapped via STRING (Figure 2c). In addition, the KEGG pathway enrichment analysis was used to map the sEV proteins affected by TSP treatment onto pathways and identify protein networks (Figure 3a). TSP‐sEVs proteins indicated that the proteasome pathway was upregulated, and glycolysis was downregulated relative to UNS‐sEV (Figure 3a). The connection network of proteins involved in upregulated and downregulated pathways was mapped using STRING (Figure 3b).

### 3.4 sEVs generated from TSP‐treated macrophages contain TSP proteins

There is evidence indicating that pathogen‐derived antigens can access the sEV packaging machinery of host MΦs (Cronemberger‐Andrade et al., 2020; Ganeshalingham & Wong, 2007). Indeed, our proteomics analysis showed that several TSP proteins are present in sEVs isolated from TSP‐pulsed MΦs, including Ras‐related protein Rab‐6B, Stress‐70 protein, ubiquitin‐conjugating enzyme E2D 2A, and other unknown proteins (Table S1). KEGG pathway analysis showed the potential pathways which the unique and shared proteins of sEVs can induce (Figure S3).

### 3.5 sEVs are captured and actively internalized by macrophages and BMDCs

**FIGURE 4:**
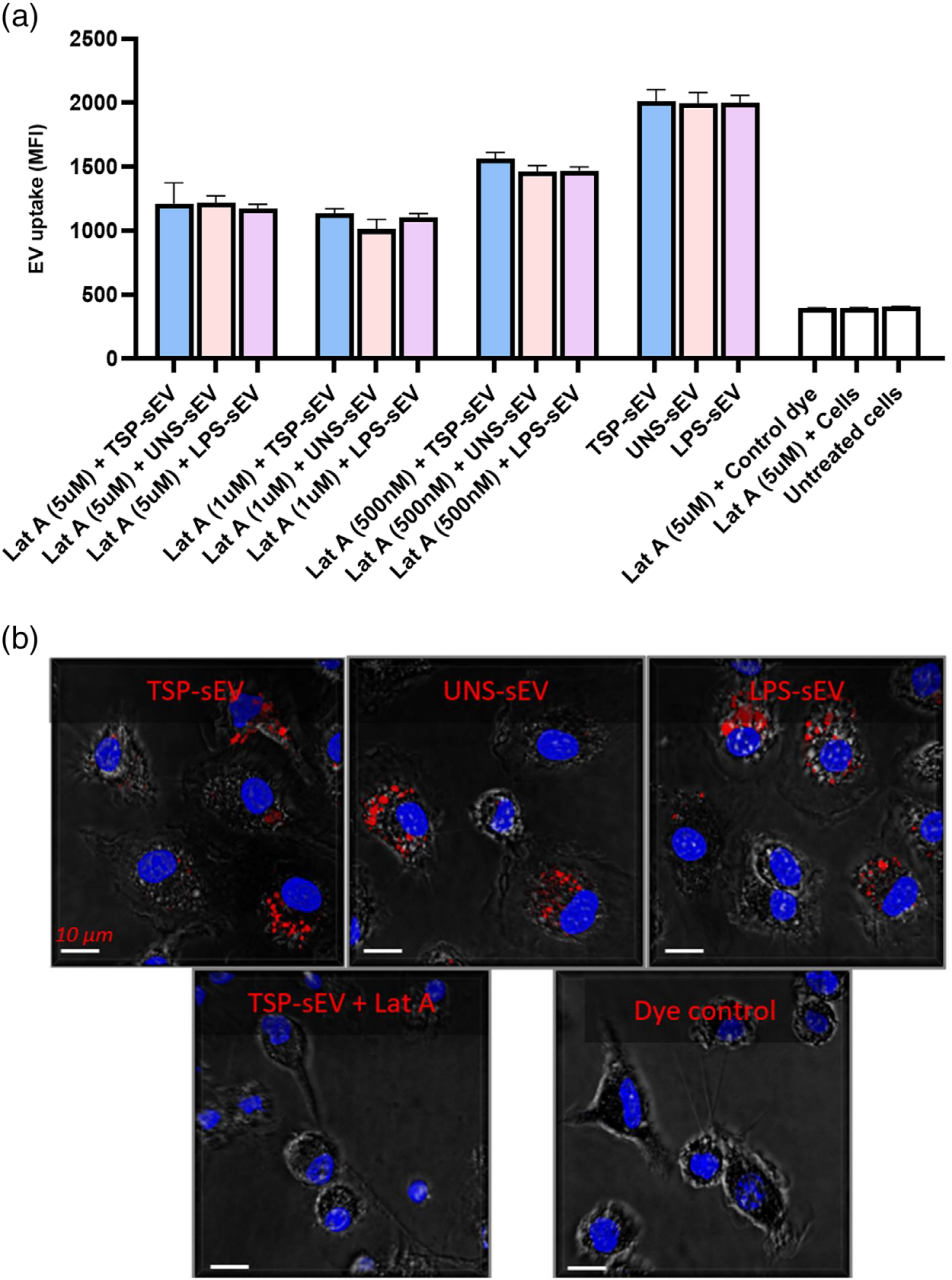
Uptake and internalisation of sEVs by BMDMs and BMDCs. (a) Assessment of sEV uptake by BMDMs following 30 min pre‐treatment with endocytosis inhibitors, Latraculin A, in a dose‐dependent manner, showed inhibition of sEV capture indicating the main process of sEV uptake is based on endocytosis. (b) Confocal microscopy images showing the internalization of PKH26 labelled sEV into naive BMDMs. Internalisation was blocked upon using Lat A as an endocytosis inhibitor. Cellular structure (gray) was visualized without fluorescence using differential interference contrast, macrophage nuclei were labelled with DAPI (blue), and PKH26‐labelled EVs were detected as red spots in the cytosol. All values are expressed as mean ± SEM of two independent experiments (triplicate)

We next examined whether sEVs from TSP‐treated MΦs are internalized by recipient immune cells. We, therefore, first determined whether sEVs can be captured and trafficked into the recipient cells (BMDMs, BMDCs, RAW cells). SEVs were labelled with PKH26 and uptake was evaluated using flow cytometry. We found that TSP‐sEVs were readily taken up by BMDMs and BMDCs with similar efficiency as those derived from LPS or unstimulated MΦs, as determined by flow cytometry (Figure S4A and S4F) and confocal microscopy (Figure 4a). Uptake was prevented at 4°C, or when endocytosis was blocked using Latraculin A, an actin polymerization inhibitor, suggesting that capture and uptake is an active process. (Figure S4B). In support, we observed pre‐treatment of recipient cells with an endocytosis blocker (Latraculin A) for 0.5 h markedly reduced sEVs uptake in a dose‐dependent manner (Figure 4b). Furthermore, chelation of calcium with ethylene glycol tetra‐acetic acid (EGTA) and saturating most C‐type lectin receptors with different sugars in recipient cells did not affect the sEVs uptake, implying sEVs were internalized via other mechanisms (data not shown). sEV uptake was confirmed using confocal microscopy where internalization of sEVs by BMDMs was visualized. The uptake of sEVs by BMDCs and RAW 264.7 macrophages was also confirmed (Figure S4C–E).

### 3.6 sEVs and lEVs derived from TSP‐treated BMDMs suppress TNF and IL‐6 production in LPS‐stimulated BMDMs and BMDCs

We next set out to test whether sEVs from TSP‐treated MΦs have immune‐modulatory potential. TSPs have been shown to have strong suppressive effects on the production of pro‐inflammatory cytokines such as TNF and IL‐6, while supporting IL‐10 production by MΦs (Hoeksema et al., 2016).

**FIGURE 5:**
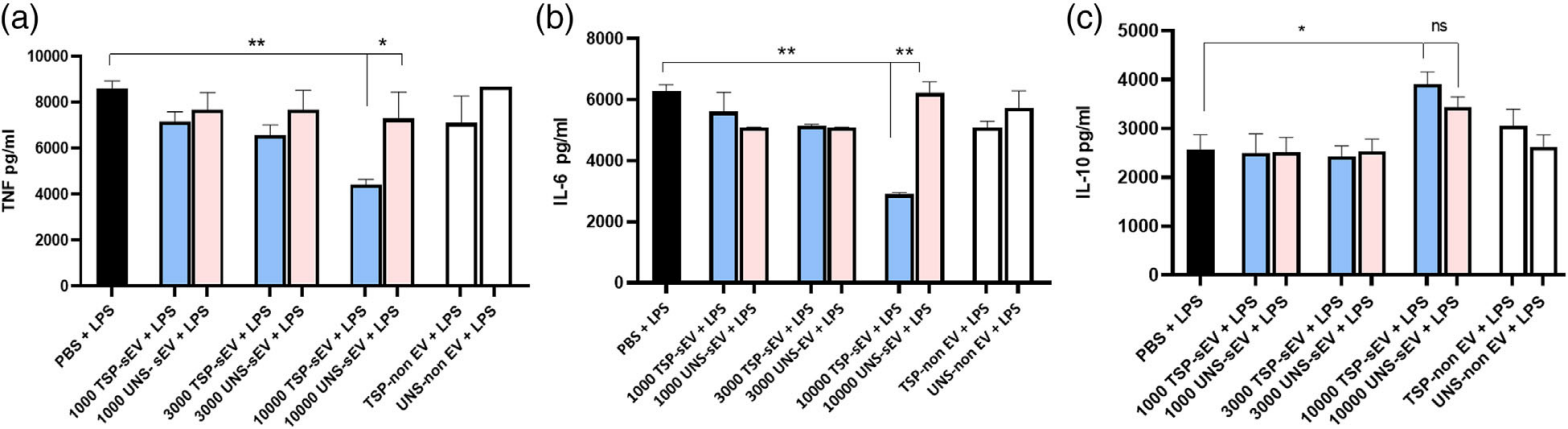
TSP‐sEVs suppress TNFα and IL‐6 from LPS‐treated BMDMs in a dose‐dependent manner. (a and b) TSP‐sEVs, but not UNS‐sEVs, could reduce TNFα and IL‐6 from LPS‐treated BMDMs in a dose‐dependent manner after 24h. sEV‐depleted groups along with PBS were included as controls. (c) IL‐10 was not significantly upregulated by TSP‐sEVs in comparison with UNS‐sEVs but shows an upregulation trend. All values are expressed as mean ± SEM of two independent experiments (triplicate). *P < .05 *P < 0.05, **P < 0.01, and ***P < 0.001

We tested whether exposure to TSP‐lEVs and TSP‐sEVs affects cytokine release from LPS‐stimulated MΦs in comparison with UNS‐lEVs and UNS‐sEVs. TSP‐sEVs, but not UNS‐sEVs, suppressed IL‐6 and TNFα production by LPS stimulated BMDMs in a dose‐dependent manner. A mild increase in IL‐10 expression was also found (Fig. 5a‐c). Similar modulatory effects on cytokine production were seen following exposure to TSP‐IEVs (Figure S5A–C). To confirm that the suppressive effect is vesicle‐mediated, non‐EV fractions along with PBS were used as an additional control. To determine if TSP‐sEVs from BMDMs can modulate BMDCs, we stimulated BMDCs with TSP‐sEVs or UNS‐sEVs in the presence of LPS. We observed a similar suppressive trend of IL‐6 and TNFα in BMDCs exposed to TSP‐sEVs (Figure S6A, B).

In support, sEVs purified (fractions 7–9) from BMDMs by SEC showed the same modulatory effect on cytokine production by BMDMs, further substantiating a role for vesicles in the findings above (Figure S7A, B).

### 3.7 TSP treatment suppresses the release of EV‐associated TNF from macrophages

Evidence suggests that TSPs can effectively suppress TNFα production in response to inflammatory agents (Hoeksema et al., 2016). As TNFα has been shown to be associated with EVs (Lebedeva et al., 2020), we investigated whether TSPs can reduce EV‐associated TNF in comparison with untreated MΦs. To this end, we assessed the concentration of TNFα in EV fractions. The collected cell culture supernatants were concentrated with Amicon filters (100 kD) and differential ultracentrifugation was performed for EV isolation, as illustrated in Figure S8B, C samples 1–9. The TNFα concentration, as measured by ELISA in all steps of sEV purification, was much reduced in TSP‐treated MΦs compared to PBS controls (Figure 8B, C). To further investigate the level of EV‐associated TNFα, sEVs were washed with PBS followed by ultracentrifugation twice and the level of TNFα in the samples was evaluated using Western blotting and ELISA. We observed that the level of TNFα in TSP‐sEVs is markedly lower than UNS‐sEVs and LPS‐sEVs (Figure S8D, E). Transcriptomic analysis showed that genes involved in the TNFα signalling pathway were significantly enriched in MΦs treated with UNS‐sEV and LPS‐sEV compared to those treated with TSP‐sEVs, in support of TSP reducing the release of sEV‐associated TNFα (Figure S8F, G). STRING was also used to map the connection of upregulated genes (Figure S8H).

### 3.8 TSP‐EVs have no effect on selected M1 marker expression

Given the suppressive effects of TSP‐sEVs on pro‐inflammatory cytokine expression, we next examined whether they also affect the expression of other markers of BMDM/DC activation. We observed that pre‐treatment with TSP‐sEVs did not affect MHCII, CD86, and iNOS expression by BMDMs both in the presence and absence of LPS relative to PBS, while UNS‐sEVs increased both MHCII and CD86 (Figure S9A). The effect of lEVs from TSP‐pulsed MΦs on M1 markers was also evaluated and showed significant suppression of iNOS relative to both PBS and UNS‐lEVs in the presence of LPS (Figure S9B). Moreover, we observed that pre‐treatment with TSP‐sEVs could decrease iNOS and CD86 expression on BMDCs relative to UNS‐sEVs (Figure S9C, D).

### 3.9 TSP‐EVs are unable to polarize naive macrophages toward M2

It has been reported that TSPs potentiate M2 polarization (Hoeksema et al., 2016) and that MΦs with an M2 phenotype release low levels of TNF and IL‐6 (Zhao et al., 2020). In this regard, IL‐4 as a potent inducer of M2 polarization mitigates the release of TNF and IL‐6 in response to LPS (Hart et al., 1991). To explore whether the suppressive effect of TSP‐sEVs on TNF and IL‐6 release from naive BMDMs is associated with polarization toward M2 phenotype, we analysed the expression of M2‐associated markers including YM‐1, CD206, RELMa, and PDL‐2 in TSP‐sEV and UNS‐sEV treated BMDMs. None of these markers were significantly induced after exposure to TSP‐sEVs in the presence or absence of LPS, indicating that the reduction in IL‐6 and TNFα production was not a reflection of polarization toward an M2 phenotype in naive BMDMs (Figure S10A, B). Similar results were observed in lEV‐treated BMDMs where no lEV type modulated M2 marker expression (Figure S10C).

### 3.10 TSP‐sEVs alter gene expression in macrophages

To further characterize the effect of TSP‐sEVs on MΦs, microarray transcriptomic analysis was performed on unstimulated and LPS‐stimulated RAW 264.7 macrophages exposed to TSP‐sEVs, LPS‐sEVs, or UNS‐sEVS. Of the 22,206 genes with an expression level above background signal around 2300 genes (fold change of more than 2 and *P*  <  0.05) were differentially expressed in MΦs treated with TSP‐sEVs, UNS‐sEVs, and LPS‐sEVs ± LPS versus PBS ± LPS (Fig 6a, b). In comparison with TSP‐sEVs and UNS‐sEVs, LPS‐sEVs showed the highest number of upregulated genes relative to PBS(Figure 6a). The number of shared and uniquely expressed genes in MΦs exposed to TSP‐sEVs, UNS‐sEVs, and LPS‐sEVs with or without LPS is shown via a Venn diagram (Fig 6c and d).

FIGURE 6Transcriptomics data analysis of macrophages exposed to TSP‐sEV and UNS‐sEV. (a and b) The total number of up‐ and downregulated genes in LPS‐treated or naive macrophages exposed to sEVs derived from TSP‐, LPS, and unstimulated macrophages. LPS‐sEV induced the highest number of upregulated genes and in turn the lowest number of downregulated genes in naive macrophages in comparison with UNS‐sEV and TSP‐sEV. UNS‐sEV could also downregulate more genes in naive macrophages relative to TSP‐sEV. (c and d) Venn diagram also showed that LPS‐sEV induced the most unique genes in comparison with UNS‐sEV and TSP‐sEV. (e) GO analysis was performed on genes expressed in macrophages exposed to TSP‐sEV versus UNS‐sEV. Top results of each representation including cellular components, biological process, and molecular function were showed as down and upregulated associated GO terms based on fold change (FC) ≥ ± 2 and *P *< 0.05. (f) KEGG pathway analysis was performed on the genes expressed by macrophages exposed to TSP‐sEV or UNS‐sEV vs PBS. No significant up and downregulated pathway was recognized in UNS‐sEV vs PBS. In contrast, analysis of genes expressed in naive macrophages after exposure to TSP‐sEV vs PBS showed three significant downregulated pathways including olfactory transduction, glutathione metabolism, and steroid hormone biosynthesis. Fold change (FC) ≥ ±1 and FDR ≤ 0.05. (g) Volcano plot of differentially expressed genes. Significantly up‐regulated genes with log2 fold change higher than two are shown as red dots and down‐regulated genes with log2 fold change more than two are shown as blue dots. Fold change (FC) ≥ ± 2 and FDR ≤ 0.05

**Figure:**
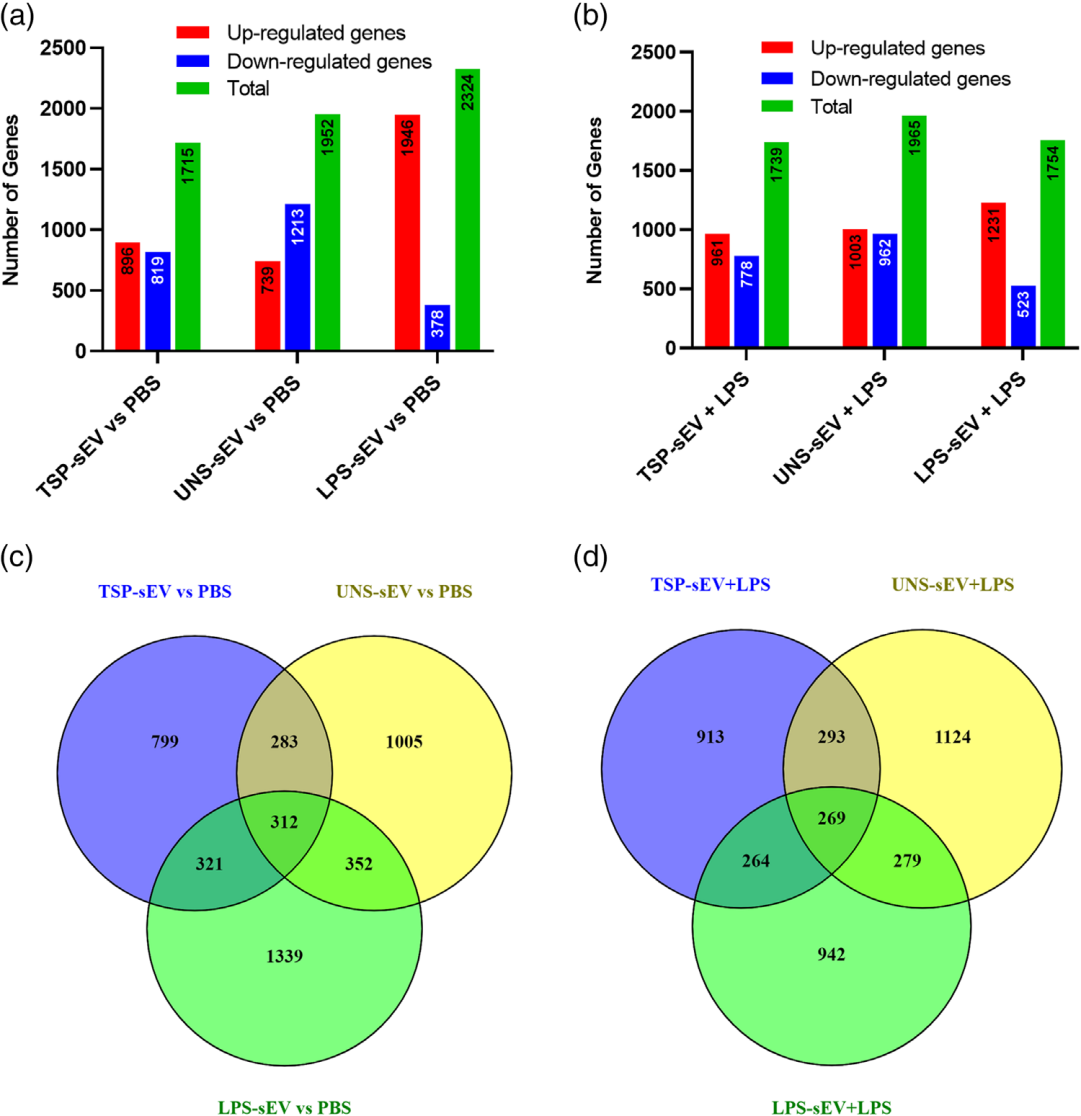

**Figure:**
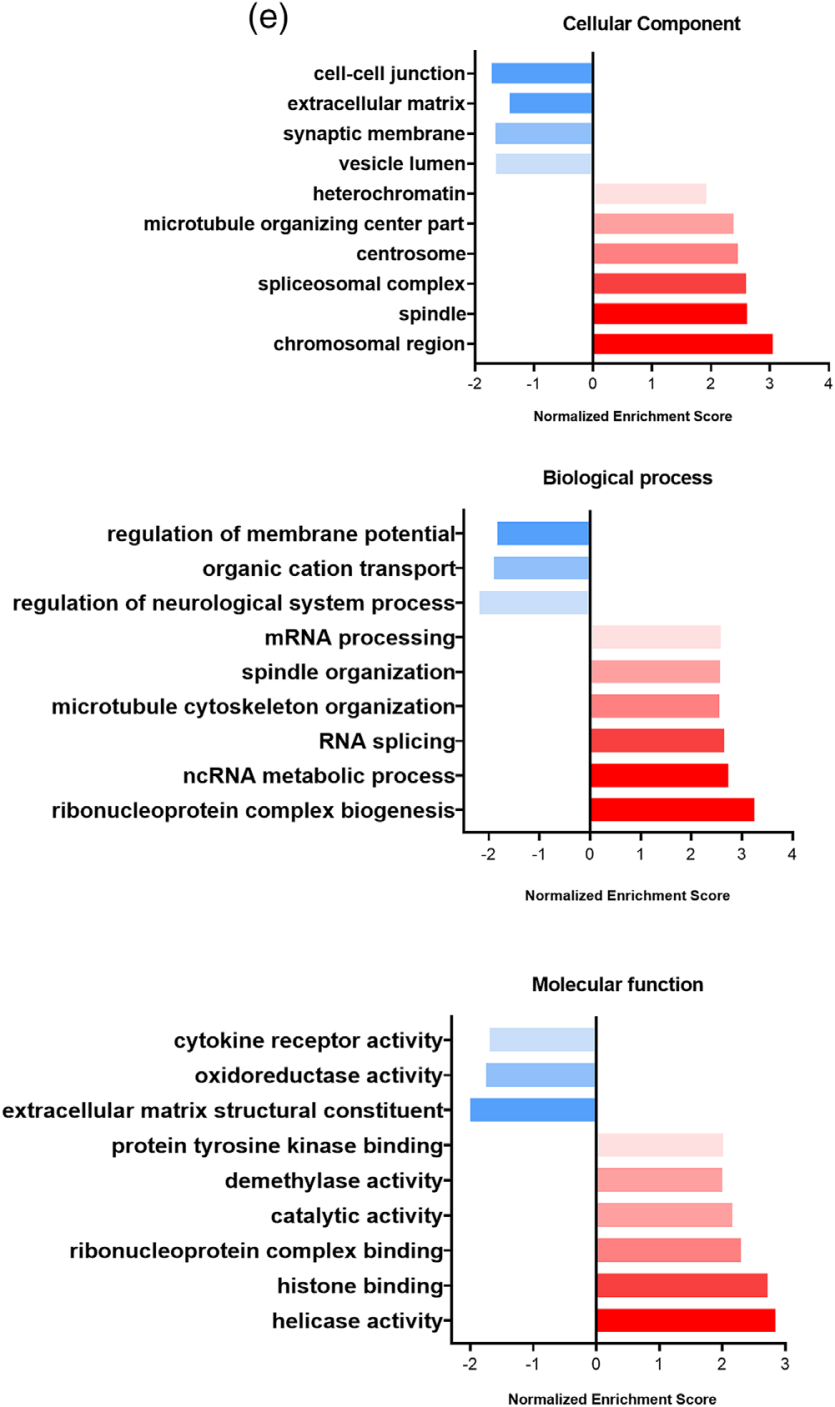

**Figure:**
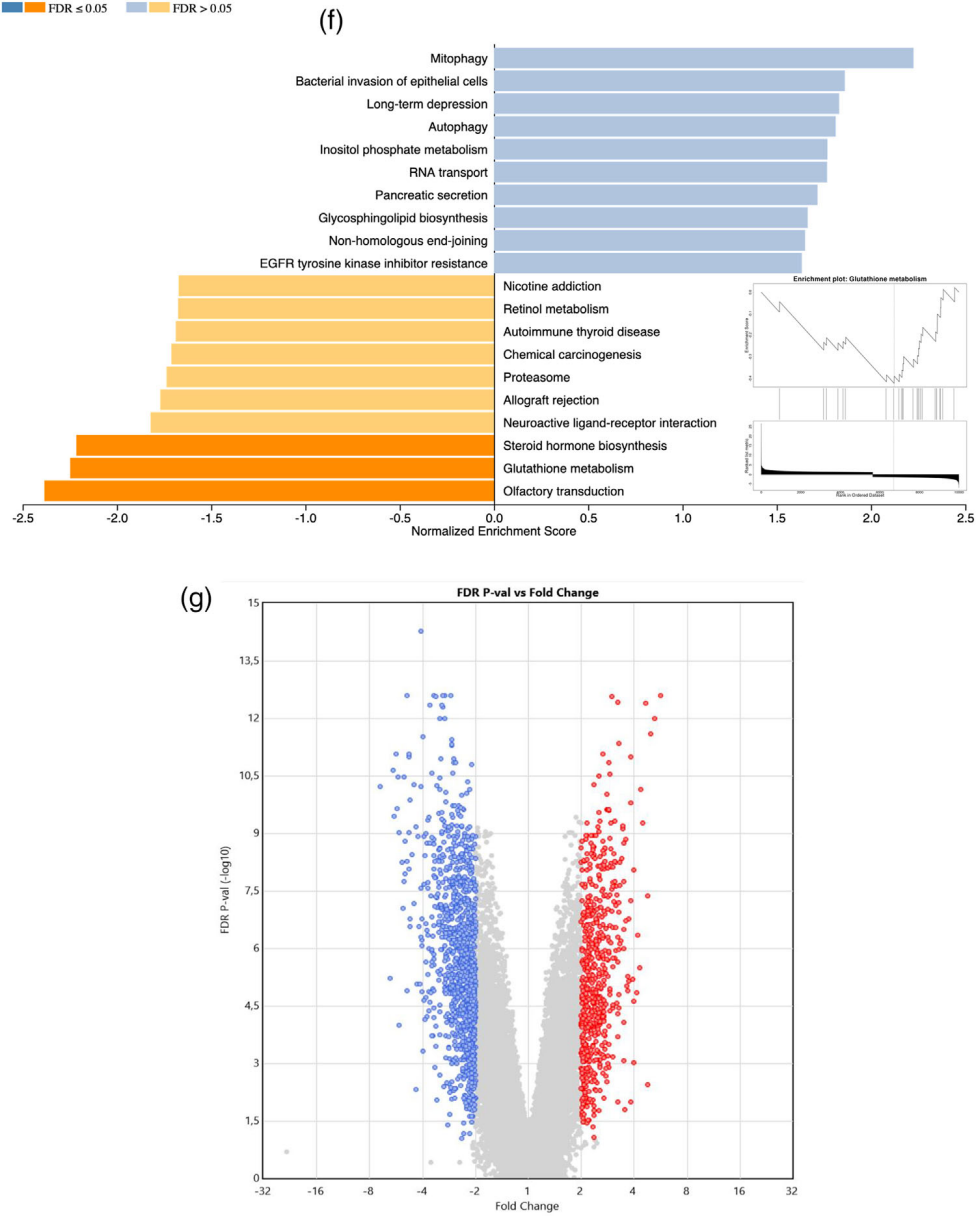

GO terms were used for annotation of the differentially expressed genes based on cellular component, molecular function, and biological process in the genes differentially expressed in TSP‐sEVs versus UNS‐sEVs treated cells (Figure 6e). Furthermore, KEGG pathway enrichment analysis was applied to identify down‐regulated and upregulated pathways. Among the significantly enriched pathways (FDR ≤ 0.05) in TSP‐sEVs versus PBS, three down‐regulated pathways were related to olfactory transduction, glutathione metabolism, and steroid hormone biosynthesis, whilst no pathway was enriched in UNS‐sEV versus PBS (Figure 6f). Genes with fold change of more than two and *P*  <  0.05 in TSP‐sEVs versus PBS are represented in a volcano plot (Figure 6g).

### 3.11 The gene expression pattern of the macrophages exposed to TSP‐sEVs reflects an altered redox and antioxidant homeostasis

**FIGURE 7:**
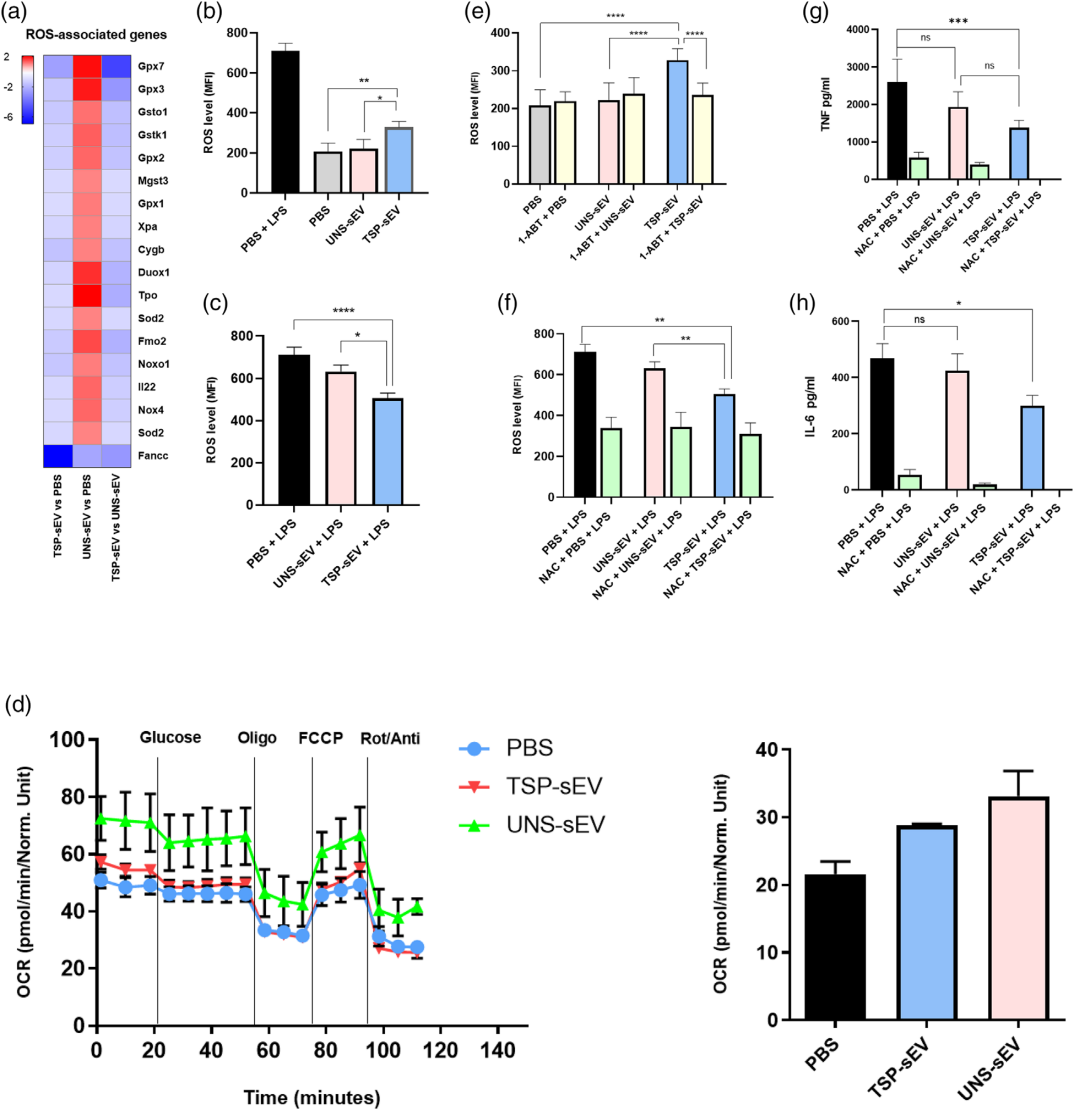
Assessment of ROS generation and associated genes in BMDMs exposed to sEVs. (a) Comparison of genes associated with ROS generation in BMDMs exposed to TSP‐sEV and UNS‐sEV showed that TSP‐sEV could suppress genes that scavenge ROS relative to UNS‐sEV, thus support ROS generation in BMDMs. (b and c) ROS level in naive BMDMs was significantly increased by TSP‐sEV relative to UNS‐sEV and PBS. In contrast, stimulation of BMDMs with TSP‐sEV in the presence of LPS could suppress ROS in comparison to UNS‐sEV and PBS. (d) Graph of OCR level after glucose, oligomycin, FCCP, and rotenone/antimycin treatment of macrophages incubated with TSP‐sEV, UNS‐sEV, and PBS for 24h. Bar graph represents the average of basal OCR calculated from graph D. (e) Pre‐treatment of TSP‐sEV with 1‐ABT (p450 inhibitor) for 30 min before adding to BMDMs could significantly abrogate ROS induction. (f) pre‐treatment of BMDMs with NAC (ROS scavenger) 30 min before stimulation with sEVs plus LPS indicated a strong suppression of ROS irrespective of sEVs stimulation. (g and h) Since NAC can strongly suppress ROS generation in BMDMs, its effect on inflammatory cytokines including IL‐6 and TNFα was measured. It showed that NAC potently inhibits the production of these cytokines. All values are expressed as mean ± SEM of three independent experiments (triplicate). *P < .05 *P < 0.05, **P < 0.01, and ***P < 0.001

Thioredoxin (TRX) and glutathione (GSH) play crucial roles in maintaining redox balance in cells by scavenging ROS (Muri & Kopf, 2021). Our transcriptomic data showed that the GSH pathway was suppressed in MΦs exposed to TSP‐sEVs in the absence of LPS compared to MΦs exposed to UNS‐sEV. In addition, we observed increased expression of genes implicated in the nuclear factor erythroid 2‐related factor 2 (NRF2) pathway that induces expression of antioxidant response genes. Many of these downstream target genes showed altered expression, some of them upregulated (Catalase, NQo1) and some were downregulated including superoxide‐dismutase, selenoprotein P, and Fanconi anaemia complementation group C (Figure 7a). This together, suggests that exposure to TSP‐sEVs alters MΦ redox and anti‐oxidant homeostasis and suggests changes in the ROS production of these cells. Indeed, exposure of MΦs to TSP‐sEVs alone was sufficient to induce ROS production, while UNS‐sEV did not (Figure 7b), although this was less in magnitude compared to cells stimulated with LPS, a well‐known driver of ROS production in MΦs (Park et al., 2015). Conversely, LPS‐ induced ROS production was suppressed by TSP‐sEVs treatment (Figure 7c).

Next, we looked for the source of ROS in TSP‐sEV treated MΦs in the absence of LPS. Cells can generate ROS as a by‐product of electron transport chain activity in mitochondria, or via activation of ROS‐producing enzymes such as iNOS and NOX. However, mitochondrial oxygen consumption rates (OCR) linked or levels of uncoupling as indirect measures for mitochondrial ROS production were not affected by TSP‐sEV treatment (Figure 7d), nor was there an increase in expression of iNOS or NOX subunits. This together may point to another source of ROS. We therefore searched for potential components transferred by TSP‐sEVs that might account for ROS induction in recipient cells. To this end, we returned to the proteomic dataset of TSP‐sEVs and looked for differentially expressed proteins with redox functions. We observed enrichments of the ROS‐inducing enzyme, CYP450 in sEVs released from TSP‐pulsed MΦs, whilst it was not detected in LPS‐sEVs or UNS‐sEVs. In accordance, there is evidence that CYP450 can be transferred between cells via EVs (Gerth et al., 2019). We then investigated whether this molecule was involved in the ROS generation by TSP‐sEVs. Since CYP450 is a heat‐sensitive protein, we pre‐heated the TSP‐sEVs to 60°C for 20 min and applied them to MΦs. Heating TSP‐sEVs could abrogate its ROS inducing effects (data not shown). Furthermore, direct blocking of CYP450 activity in sEVs using the P450 selective inhibitor 1‐Aminobenzotriazole (1‐ABT) (Figure S11) blunted their ability to induce ROS production in MΦs, suggesting a key role for EV‐derived CYP450 in driving ROS production in MΦs (Figure 7e).

Since LPS‐driven pro‐inflammatory cytokine expression is known to be supported by induction of ROS, derived from mitochondria (Park et al., 2015), the ability of TSP‐sEVs to suppress LPS‐driven IL‐6 and TNFα expression might be due to impaired mitochondrial ROS production by these cells. In addition, TSP‐sEVs failed to suppress TNFα and IL‐6 in BMDMs exposed to intracellular TLR agonists such as R848 (TLR7/8) and CpG ODNs (TLR9 agonists) (data not shown). This might imply that TSP‐sEVs block LPS or mask TLR4 and prevent interaction with LPS. Moreover, scavenging of ROS using N‐acetyl cysteine (NAC) (Figure 7f) was sufficient to reduce LPS‐induced IL‐6 and TNF expression (Fig 7g, h). Together, this suggests that TSP‐sEVs suppress pro‐inflammatory cytokine production by preventing mitochondrial ROS release.

### 3.12 Seahorse metabolic pathway analysis on BMDM exposed to sEVs and lEVs

Since we observed a significant induction of ROS generation by TSP‐sEVs, we aimed to examine whether this capacity causes metabolic reprogramming in MΦs. To do so, we conducted a Seahorse XF analysis on BMDMs which had been exposed to sEVs and lEVs derived from TSP‐treated or unstimulated MΦs. We measured alterations in the oxygen consumption rate (OCR) and extracellular acidification rate (ECAR) in BMDMs exposed to sEVs and lEVs. Our results showed no significant difference in OCR and ECAR level in BMDMs exposed to sEVs and lEVs derived from TSP‐treated or unstimulated MΦs (Figure S12).

## 4 DISCUSSION

The parasitic worm, *T. suis*, has been shown to support the regulatory phenotype of host antigen presenting cells (APCs) via induction of IL‐10 and suppression of TNFα and IL‐6 (Ottow et al., 2014). Although the immunomodulatory effects of *T. suis‐*derived antigens such as TSPs have been studied, it was unknown whether TSPs could condition MΦs to release regulatory EVs. In fact, our research rationale originated from investigations reporting that EVs released from MΦs exposed to anti‐inflammatory factors possess regulatory functions. Based on these findings, we reasoned that EVs released by TSP‐pulsed MΦs might suppress inflammatory responses in recipient cells.

In the present study, the typical shape of the vesicles was confirmed using TEM and distinct peaks in NTA were also showed that the isolated particles were in the size range of small EVs (50–150 nm). The size of the lEVs was also in accordance with the range of lEVs reported by previous studies (Tkach et al., 2017). Isolated vesicles were further characterized by detecting canonical markers harbouring by sEVs, including CD63, CD9, HSP90, and ALIX. Altogether, the results showed that the observed outcomes were primarily due to EV function, not associated debris.

Some studies have demonstrated that the capability of cells for EV generation depends on cell activation status. For instance, activated RAW264.7 macrophages released more vesicles than unstimulated control cells (Shi et al., 2020). Our findings also showed that pulsing RAW264.7 macrophage cells with LPS increased EV release. However, we observed that LPS‐treated BMDMs released fewer sEVs than naive BMDMs. This result has also been reported for BMDCs in which LPS suppressed EV generation relative to immature DCs (Segura et al., 2005; Théry et al., 1999). In another cell line, AC16 human cardiomyocytes, LPS stimulation was also found to decrease EV production (Bell et al., 2019).

This work has shown plasticity in the cargo of sEVs of macrophages and that pro‐ or anti‐inflammatory stimuli can significantly alter protein loading within sEVs. Indeed, TSP stimulation of MΦs resulted in extensive changes to the sEV proteome with 693 unique proteins identified in TSP‐sEVs. Importantly, 431 proteins were common to UNS, LPS, and TSP‐sEVs. GO analysis revealed enrichment of threonine‐type peptidase activity that was confirmed by enrichment of proteasome pathways in KEGG analysis. Likewise, GO term, RNA binding was enriched in common EV proteins with STRING analysis, displaying enrichment of the polysome network. The presence of these proteins regardless of activation state or stimuli suggests a core network of proteins in MΦ derived sEVs and may present a homeostatic role for the EV mediated transfer of RNA binding proteins and proteasome subunits. Of note, these pathways were further enriched in sEVs from TSP treated MΦs.

Previous investigations have reported that EVs released from MΦs infected with viruses and bacteria contain pathogen‐derived proteins (Giri et al., 2010; Pelchen‐Matthews et al., 2004) and the immunological effects of EVs have partly been ascribed to microbial proteins sorted in EVs (O'neill & Quah, 2008). We showed that several TSP proteins are sorted into sEVs from TSP‐pulsed MΦs but how these proteins gained access to the EVs is currently unclear. Two main pathways for protein sorting into EVs have been suggested, including ESCRT (endosomal sorting complex required for transport)‐dependent machinery which recognizes ubiquitination proteins and the ESCRT‐independent via aggregation of membrane proteins supporting EV membrane domain (Margolis & Sadovsky, 2019). Alternative pathways such as phagosome‐mediated delivery of proteins into EVs have also been indicated but require further elucidation (Margolis & Sadovsky, 2019). However, we did not assess protein ubiquitination or other potential pathways, and further investigations are required to identify the mechanisms by which TSP proteins are sorted into EVs.

Many studies have already shown that sEVs can be captured by recipient cells via various mechanisms dependent or independent of clathrin. Clathrin‐independent mechanisms include caveolin‐mediated uptake, macro‐pinocytosis, phagocytosis, and lipid raft‐mediated internalization (Mulcahy et al., 2014). In this study, we tracked the uptake of sEVs and visualized their internalization using confocal microscopy. Consistent with prior studies, blocking endocytosis using Latrunculin A significantly impaired the uptake of sEVs by recipient cells in a dose‐dependent manner, implying that endocytosis is one of the primary mechanisms by which these sEVs gain access to the cytosol (Eitan et al., 2017). Furthermore, we demonstrated that EV uptake is an active and temperature‐sensitive process, as the recipient cells could not internalize the sEVs at 4°C (Kuipers et al., 2020). Given the suppressive effects we observed with TSP‐sEVs but not with UN‐sEVs, it was important to show that TSP‐sEVs are captured and internalized with a comparable efficiency and amount as UNS‐sEVs. This would indicate that the observed effects are not due to difference in sEV uptake.

Several studies have shown that TSPs exert strong immunosuppressive effects via different mechanisms, including inhibition of TLR4 signalling and induction of regulatory cytokines such as IL‐10 and TFGβ in human monocytes (Kooij et al., 2015). Interestingly, similar to TSPs we observed that TSP‐sEVs and IEVs suppressed the production of cytokines TNFα, IL‐6, and IL‐10 in both LPS‐stimulated MΦs and BMDCs. Whether the immunoregulatory effects of TSP‐sEVs, IEVs, and TSPs relate to the shared proteins warrants further investigation.

Previous studies have reported that some cytokines such as TNFα and TGFβ can be encapsulated within or bound to EVs and thereby drive inflammation or immunoregulation in recipient cells (Barnes & Somerville, 2020). We found that TSP‐sEVs contained lower levels of TNFα than LPS‐ or UNS‐sEVs, indicating TSP treatment prevented TNFα transfer to sEVs. In accordance, the expression of genes involved in TNFα signalling was downregulated in MΦs exposed to TSP‐sEVs compared to UNS‐sEVs. However, we have not addressed the mechanisms behind the prevention of EV‐associated TNFα, but unravelling the potential pathway could be an interesting inquiry for future studies.

Bouchareychas *et al*. (2020) reported that sEVs from IL‐4‐treated BMDMs increased the expression of M2 marker genes such as arginase‐1 and RELMα and suppressed TNFα and IL‐1β expression compared to PBS in MΦs. However, no difference was observed between the functionality of sEVs from unstimulated BMDMs and IL‐4‐treated BMDMs (Bouchareychas et al., 2020). We speculated whether sEVs from TSP‐treated MΦs can also affect M1 and M2 markers. In contrast with Bouchareychas *et al*., we observed that UNS‐sEVs did not affect M2 marker levels (RELMα, YM‐1, and PDL2) yet increased levels of CD86 and MHCII M1 markers, indicating the capacity of UNS‐sEVs in activating recipient MΦs. Conversely, we observed that MHCII and CD86 were downregulated by TSP‐sEVs compared with UNS‐sEVs whilst none of the analysed M2 markers were affected. Combined, this suggests that the suppressive effects of TSP‐sEVs on inflammatory cytokines are not due to MΦ polarization. To our knowledge, this is the first time that the effect of sEVs from MΦs treated with worm‐derived antigens on MΦ polarization has been investigated.

An intriguing finding was that TSP‐sEVs were able to promote ROS production in recipient cells. Based on proteomic analysis of sEVs we identified the presence of the enzyme CYP450 as a potential source for ROS induction and its pharmacological inhibition significantly impaired their capacity to elevate ROS in naive recipient MΦs. Interestingly, it has been reported that CYP450 can be transferred by EVs and delivered to recipient cells where they modulate the redox system (Gerth et al., 2019; Rahman et al., 2019). Indeed, the gene expression profile of recipient MΦs was characterized by a reduced glutathione pathway and altered expression of genes linked to anti‐oxidant pathways (Lambeth, 2004).

It is the first time we report that sEVs from TSP‐treated MΦs can upregulate ROS in naive MΦs and present EV‐derived CYP450 as a candidate ROS inducer in recipient cells. Further investigations are required to determine whether other factors (in addition to CYP450) in TSP‐sEVs may affect ROS production or reduction of GSH. Moreover, the functional importance of this effect is currently unclear. Previous studies have reported that early infection with parasitic worms generates NO and H_2_O_2_ production by eosinophils and MΦs in the peritoneal cavity (Abd Ellah, 2013; Jedlina et al., 2011). Targeting the redox system in favor of ROS induction might therefore be one of the mechanisms by which worms accelerate the repair of damage (Dunnill et al., 2017). Thus, future studies could define whether infection with live worms can upregulate ROS production in intestinal epithelial cells.

Finally, we observed that TSP‐sEVs suppressed LPS induced ROS production, which may be a mechanism through which TSP‐sEVs suppress pro‐inflammatory cytokine production. This suggests that TSP‐sEVs, on the one hand, can promote ROS production via the release of ROS‐producing enzymes while also interfering with LPS induced ROS production in recipient cells. Whilst expression levels of TLR4 were unchanged between TSP, UNS and LPS‐sEV treated cells, EV mediated blocking of LPS interactions with TLR4 or downstream pathways is possible. What factors released by TSP‐sEVs or upstream signalling pathways mediate this effect will be a subject of further research.

It is well‐known that the primary metabolic pathway in M1 MΦs is aerobic glycolysis, while oxidative phosphorylation is activated in M2 MΦs, which increases respiratory capacity (O'neill et al., 2016). Assessment of metabolic pathways in MΦs exposed to sEVs showed no significant difference in OCR and ECAR in recipient cells, indicating none of the main metabolic pathways are targeted by sEVs.

Several questions remain unanswered at present. Characterizing the miRNA profile of TSP‐sEVs may unravel their potential targets and may in part explain their suppressive effects in recipient cells. Another potentially fruitful avenue for future research is identifying the mechanistic pathways by which parasite antigens affect the EV machinery of host immune cells. Whether helminths distribute their antigens via EVs and develop systemic immunoregulation would be a novel topic for investigation in the field of immunoparasitology. Likewise, our findings are limited to *in vitro* assessments; however, including an *in vivo* model may offer further, immunological insights.

To conclude, we observed that TSPs could profoundly modulate MΦs‐derived sEVs both in terms of functionality and biological cargo. Importantly, TSP‐sEVs induced anti‐inflammatory effects on recipient MΦs without shaping MΦs toward M2, but likely via modulation of the redox system. Modulation of host EV machinery by TSPs provides another potential mechanism by which worms can manipulate and exploit host immune cell‐cell communication to the desired setting and potentially amplify anti‐inflammatory signals throughout host cells. Future studies will be needed to show whether parasitic worms can affect the sEVs machinery of MΦs *in vivo* to suppress inflammatory responses.

## CONFLICT OF INTEREST

None.

## AUTHORS CONTRIBUTION

Amin Zakeri performed experiments, collected data, and wrote the manuscript. Amin Zakeri, Peter Nejsum, and Bart Everts contributed to designing the experiments and interpreting the results. Bradley J. Whitehead provided guidance and assistance in RNA isolation, Western blot, and transcriptomics analysis. Allan Stensballe conducted the proteomics assay and Clarize de Korne performed fluorescence microscopy. Andrew R. Williams provided parasite antigens and contributed to revising the manuscript. Amin Zakeri and Peter Nejsum conceived and defined the project. Peter Nejsum and Bart Everts supervised the project and revised the manuscript.

## Supporting information

Supplementary information
