# Supplementary material for: Parasite worm antigens instruct macrophages to release immunoregulatory extracellular vesicles
Source: J Extracell Vesicles. 2021 Aug 16;10(10):e12131. doi: 10.1002/jev2.12131 (PMC8365858; doi:10.1002/jev2.12131)
Supplement: Supplementary file 1 — Supplementary information [file JEV2-10-e12131-s001.docx]

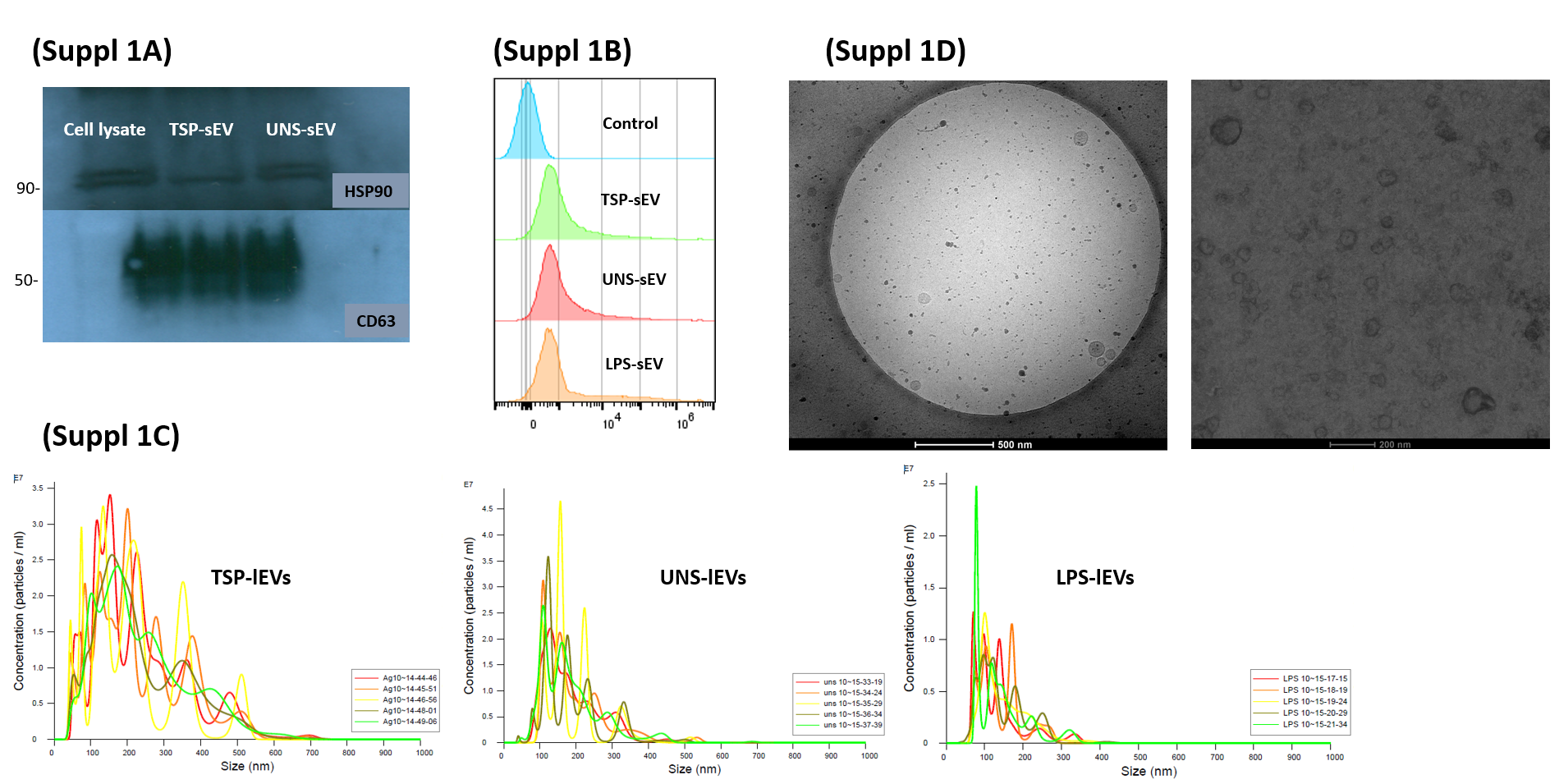


**Suppl Fig 1. ‎**Characterization of macrophage sEV and lEV NTA.

**(A)** Western blot analysis of sEV markers CD63 and HSP90 in BMDM-sEV and cell lysate (representative of two independent experiments). **(B)** Detection of CD9 marker on sEV derived from BMDM-pulsed with TSP using flow cytometry (representative of two independent measurements) **(C)** NTA data showing size and concentration distribution of lEVs derived from BMDMs pulsed with TSP, LPS, or without stimulation after a 24 h period of culture. **(D)** Cryo-EM and TEM micrograph of sEVs from BMDM cells.


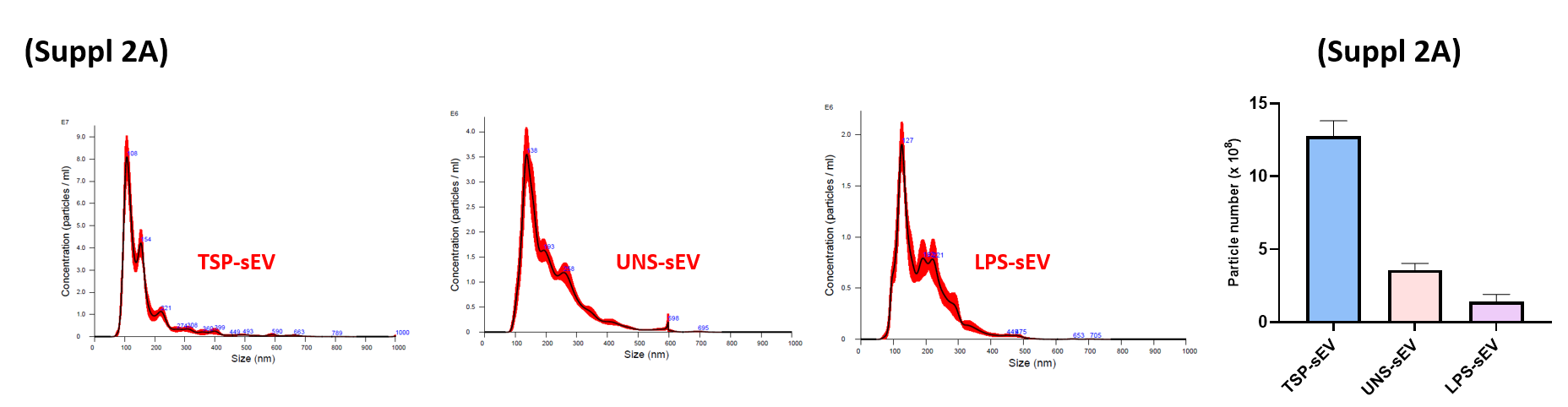


**Suppl Fig 2**. Comparison of sEV generation by different stimuli in BMDMs.

**(A)** NTA data showing size and concentration distribution of sEV derived from BMDMs pulsed with TSP, LPS, or without stimulation after a 24 h period of culture. **(B)** Showing a graph comparison sEV numbers released by BMDMs exposed to TSP, LPS or left unstimulated after 24h (representative of two independent measurements).


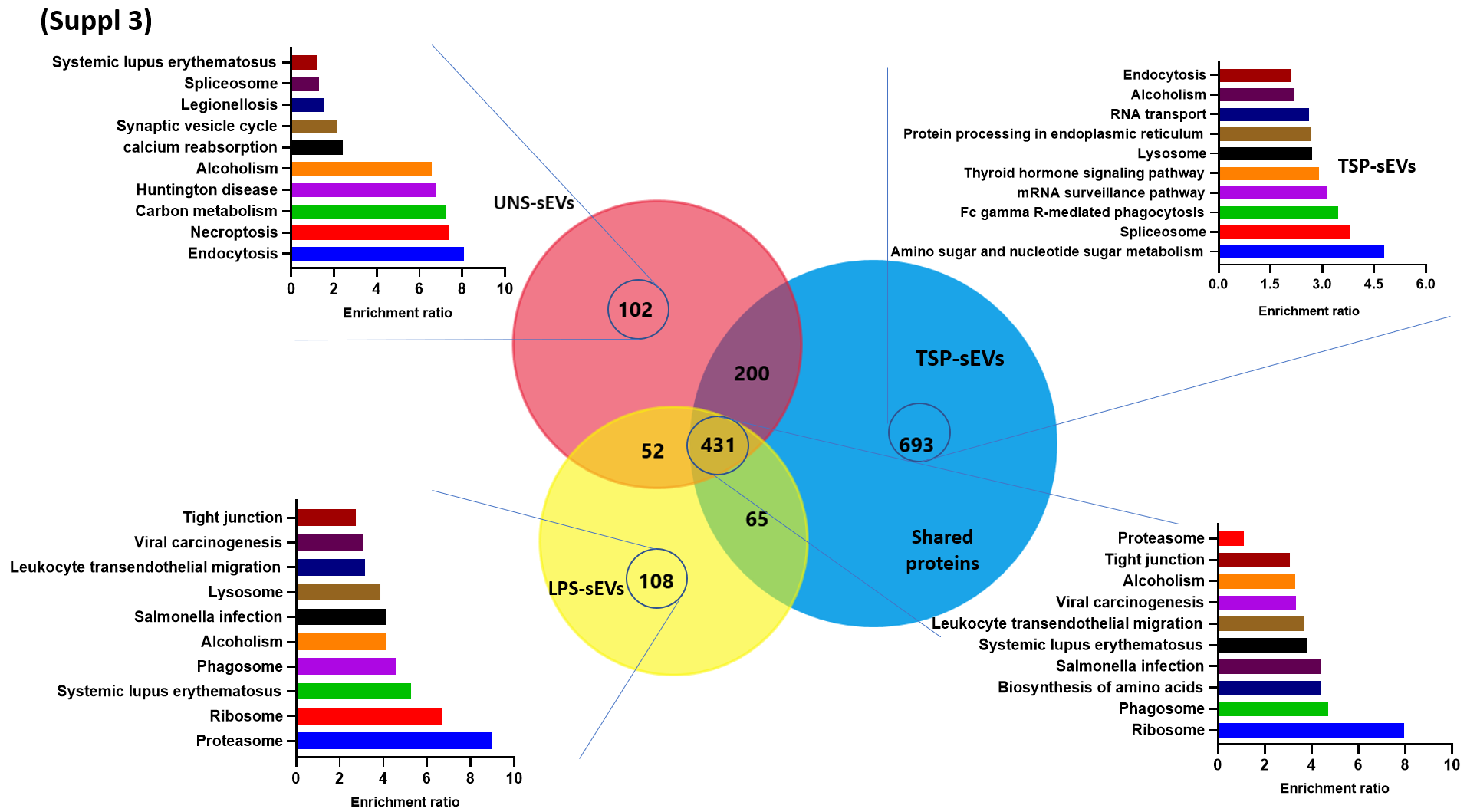


**Suppl Fig 3**. Common and unique proteins found in the three groups of sEVs (TSP-sEV, UNS-sEV, and LPS-sEV) by LC-MS/MS.

Unique proteins of each sEV group were identified and analysed with over-representation method via KEGG pathway database. Most of proteins in TSP-sEV, UNS-sEV, and LPS-sEV were involved in sugar metabolisms, endocytosis, and proteasome, respectively. In addition, 415 proteins were identified as shared between all sEVs which mostly contribute to ribosome and phagosome (FDR ≤ 0.05).


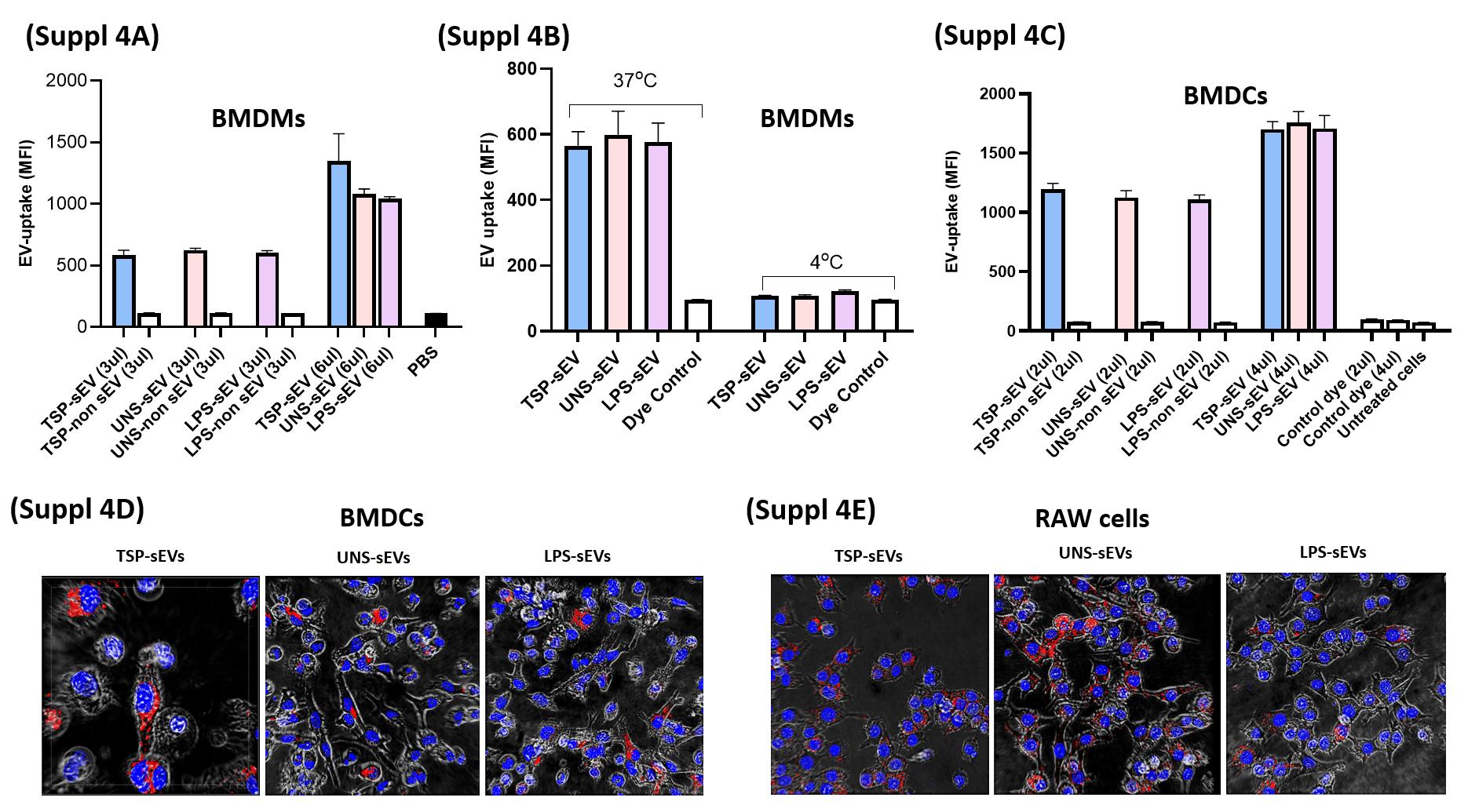


**Suppl Fig 4**. Uptake and internalisation of sEVs by BMDMs and BMDCs.

**(A)** Dose-dependent uptake of PKH-26 labelled sEVs from TSP, LPS-pulsed and unstimulated macrophages by naive BMDMs using flow cytometry. BMDMs were exposed to 3ul or 6ul of labelled sEVs for 2h in incubator at 37 °C (representative of two independent measurements). **(B)** Temperature-dependent uptake of sEV by BMDM in which exposure of recipient cells on ice abrogated their ability to capture labelled-sEV while incubating at 37 °C enabled them to uptake sEVs. **(C)** Capturing PKH26-labelled sEVs from TSP, LPS-pulsed and unstimulated macrophages in a dose-dependent manner by naive BMDCs using flow cytometry. BMDCs were exposed to 3ul or 6ul of labelled sEVs for 2h in incubator at 37 °C. (representative of two independent measurements). **(D)** Confocal microscopy images showing the internalization of PKH26 labelled sEVs into naive BMDCs and RAW cells. Cellular structure (gray) was visualized without fluorescence using differential interference contrast, cell nuclei were labelled with DAPI (blue), and PKH26-labelled sEVs was detected as red spots in the cytosol.


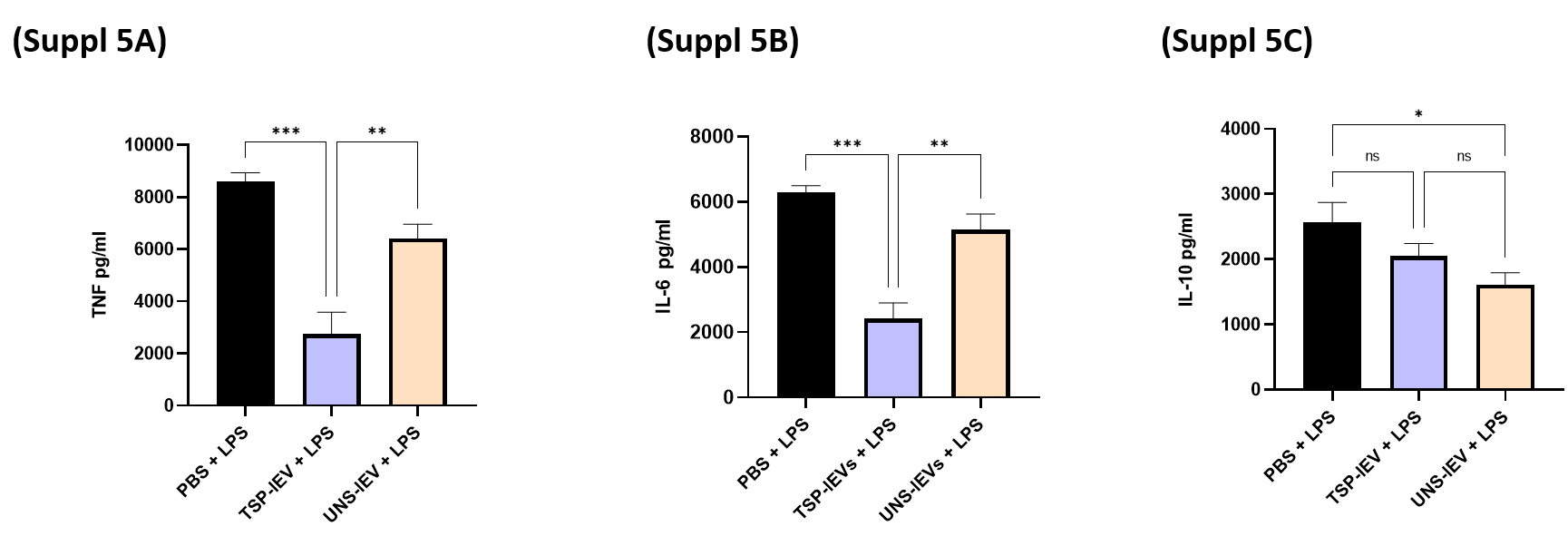


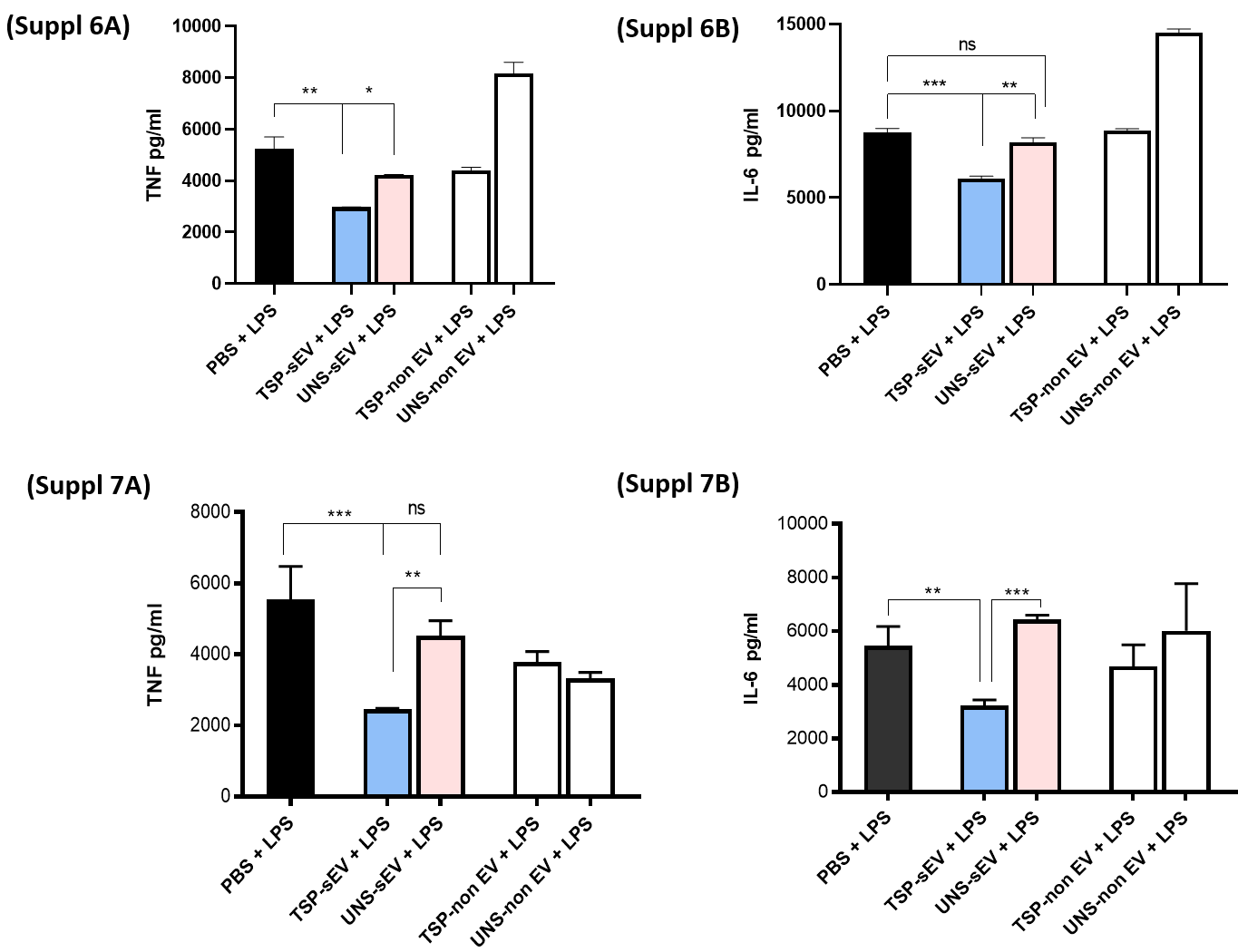


**Suppl Fig 5**. TSP-lEVs suppress TNFα and IL-6 from LPS-treated BMDMs.

**(A-C)** Suppression of inflammatory cytokines (TNFα and IL-6) was shown by using 2500 lEVs per recipient cells in the presence of LPS (10ng/ml). All values are expressed as mean ± SEM of two independent experiments (triplicate). *P < .05 ‎*p < 0.05, **p < 0.01, and ***p < 0.001‎

**Suppl Fig 6**. Suppression of inflammatory cytokines in BMDCs by TSP-sEVs.

Stimulation of LPS-treated BMDCs with 10^4^ TSP-sEV or UNS-sEV per recipient cell showed that TSP-sEV could significantly suppress TNFα **(A)** and IL-6 **(B)** in comparison with sEV-derived from unstimulated macrophages. All values are expressed as mean ± SEM of two independent experiments (triplicate). *P < .05 ‎*p < 0.05, **p < 0.01, and ***p < 0.001‎

**Suppl Fig 7**. TSP-sEVs isolated by size exclusion chromatography (SEC) method decrease inflammatory cytokines.

LPS-treated BMDMs were exposed to 10^4^ TSP-sEVs or UNS-sEVs per recipient cell and the level of TNFα (A) and IL-6 (B) was assessed using ELISA. All values are expressed as mean ± SEM of two independent experiments (triplicate). *P < .05 ‎*p < 0.05, **p < 0.01, and ***p < 0.001‎


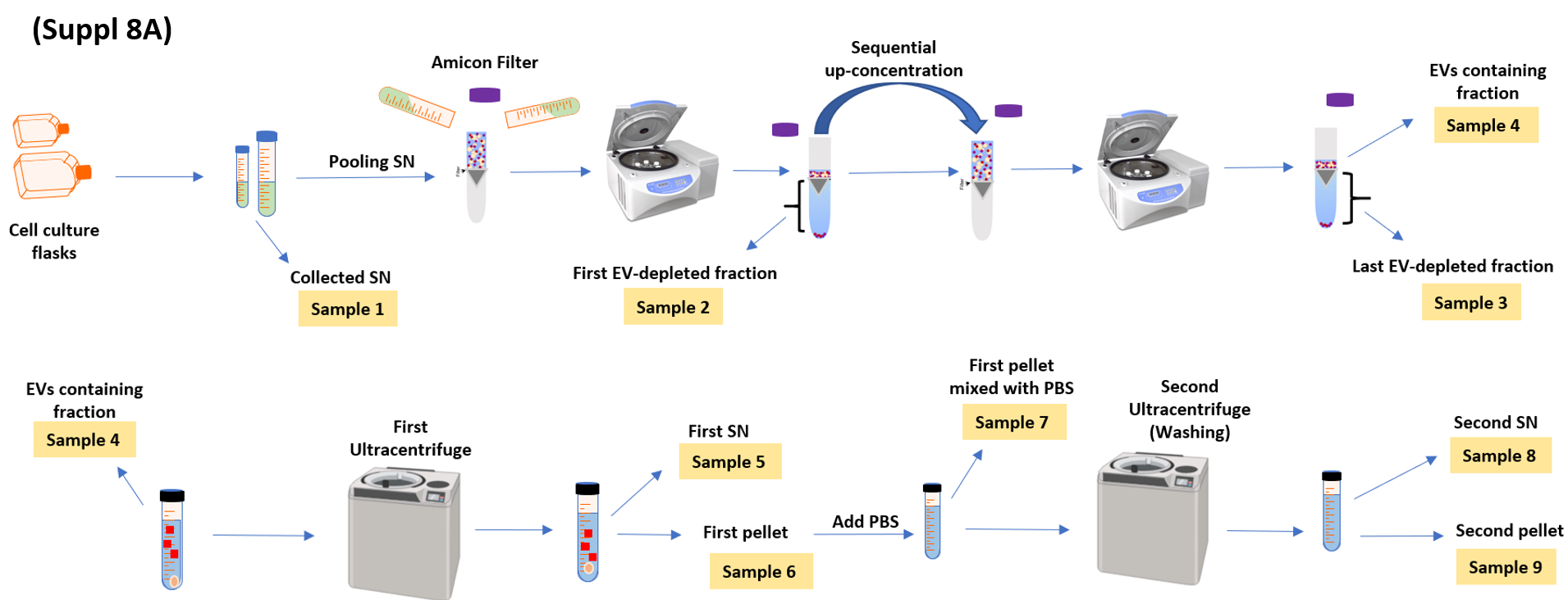


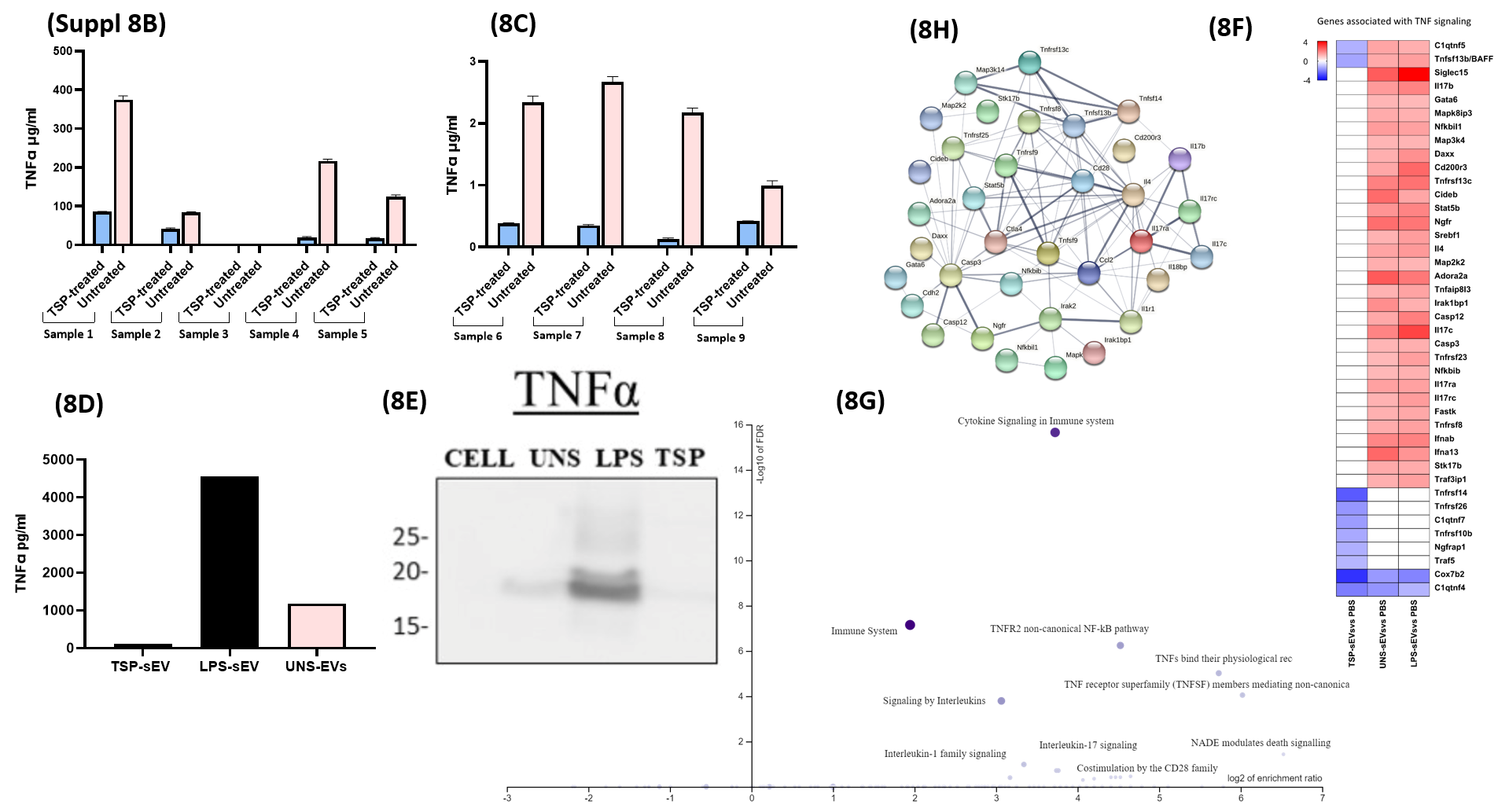


**Suppl Fig 8**. Presence of sEV-associated TNFα in sEV derived from RAW 264.7 macrophages.

**(A)** Schematic illustration of culture media up-concentration and sEV isolation via ultracentrifugation. Each step yielded a sample used for TNFα detection. **(B and C)** The level of TNFα was measured in each sample both in TSP-pulsed and unstimulated macrophages, showing that TNFα is much higher in samples derived from unstimulated macrophages. (representative of two independent measurements) **(D and E)** sEVs from LPS-stimulated macrophages were included as positive control showing that TNFα is detectable after lysing sEVs with RIPA buffer and measuring using ELISA (D) or Western blot (E). **(F)** Transcriptomics analysis showing upregulation of genes involved in TNF signaling in macrophages exposed to UNS-sEV and LPS-sEV relative to TSP-sEV treated macrophages. **(G and H)** STRING analysis illustrated the interaction between molecules involved in TNF signaling induced by UNS- and LPS-sEV. KEGG pathway analysis further indicated that TNF signaling is significantly enriched in macrophages treated with UNS- and LPS-sEV.


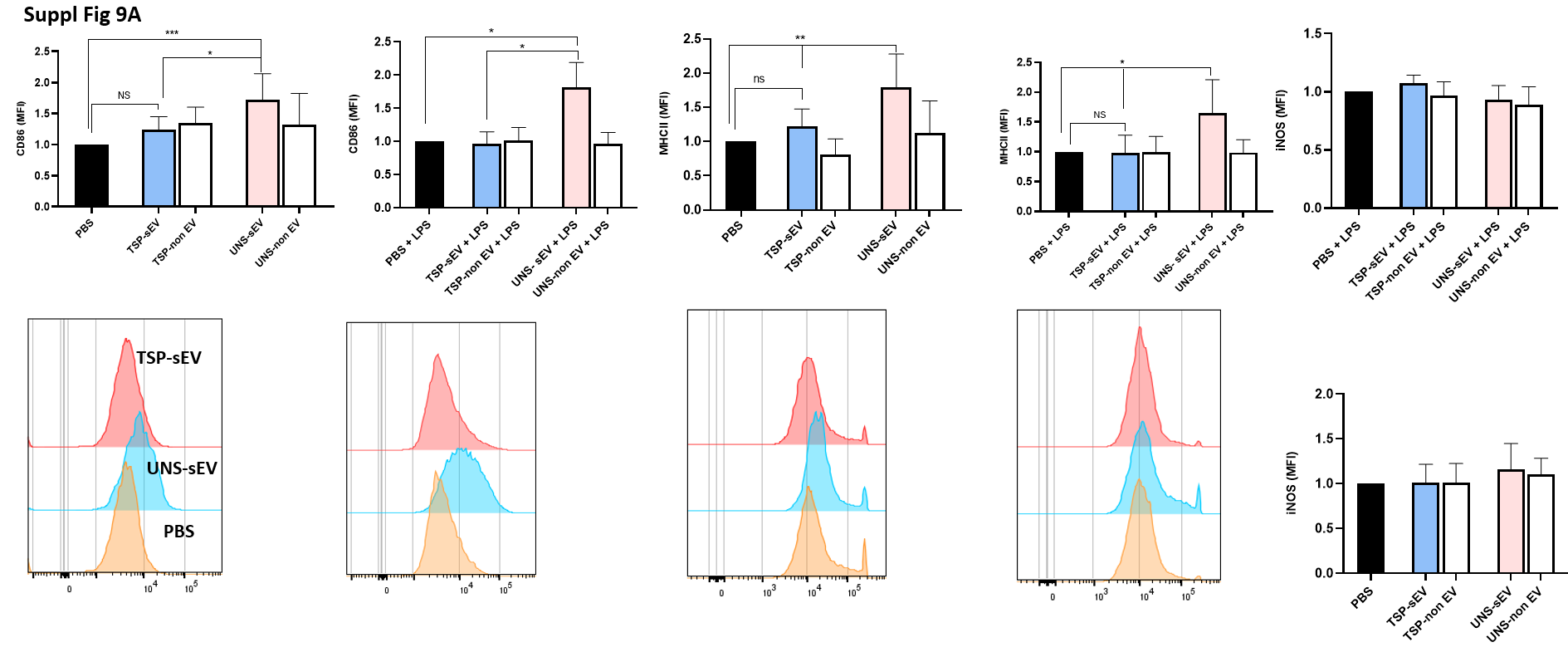


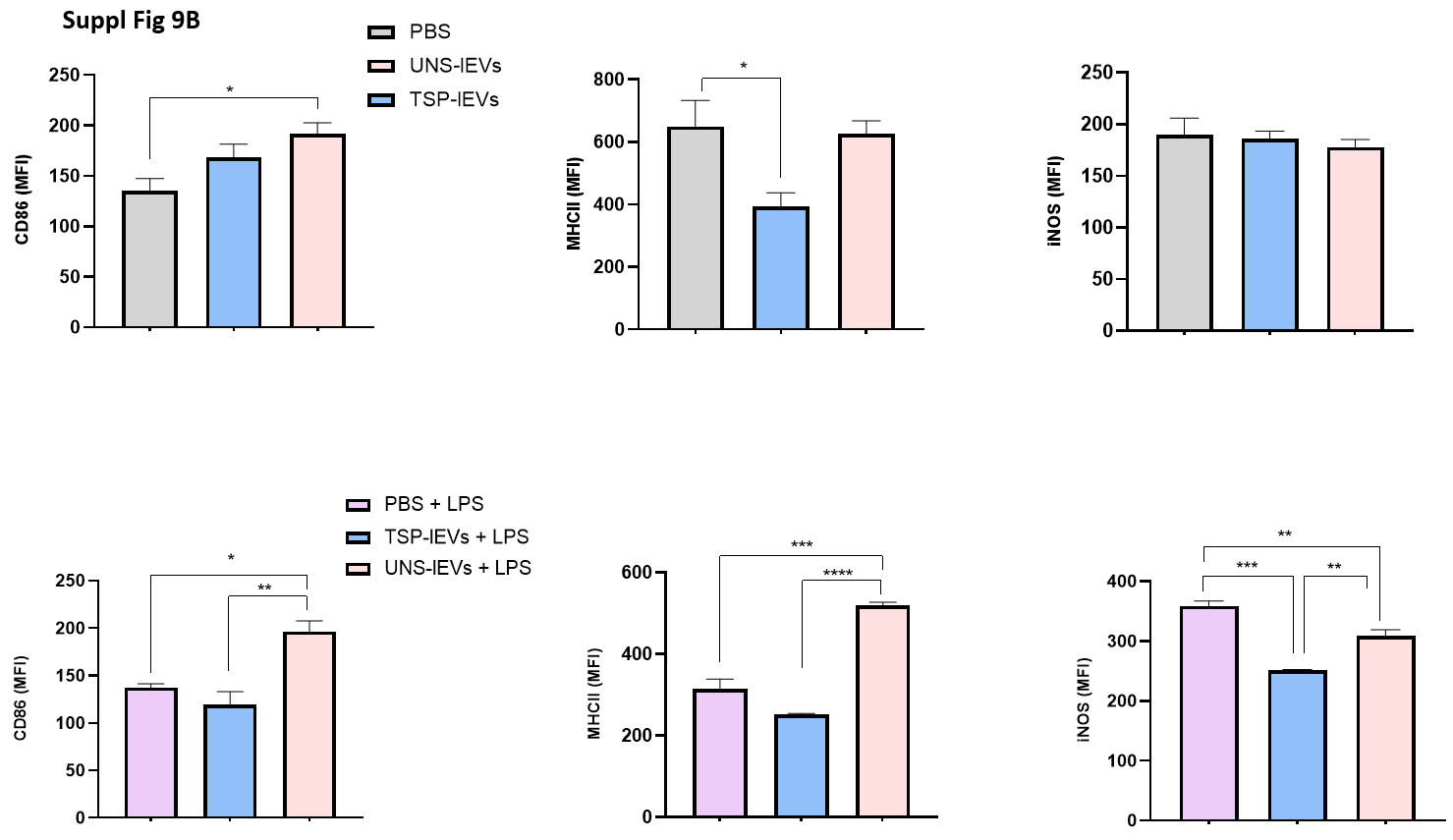


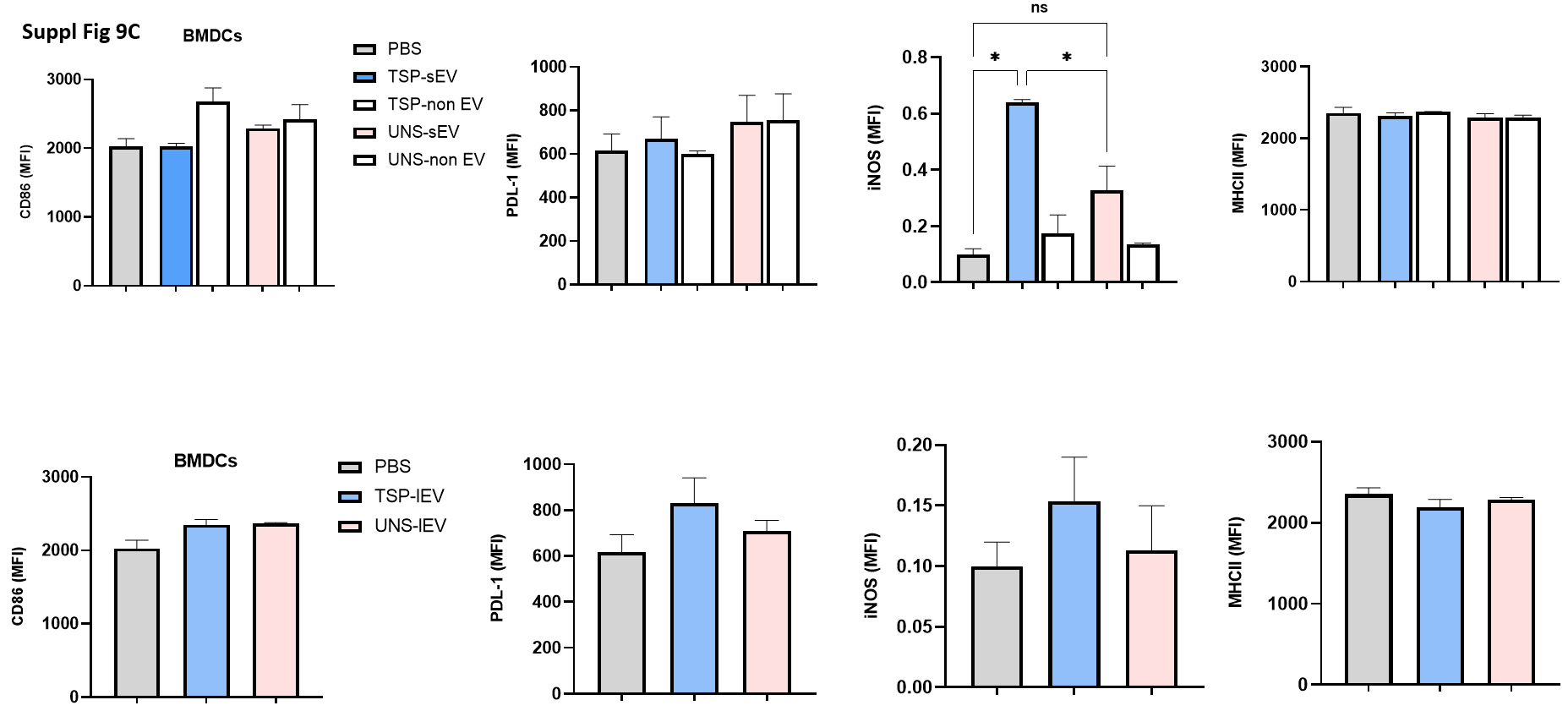


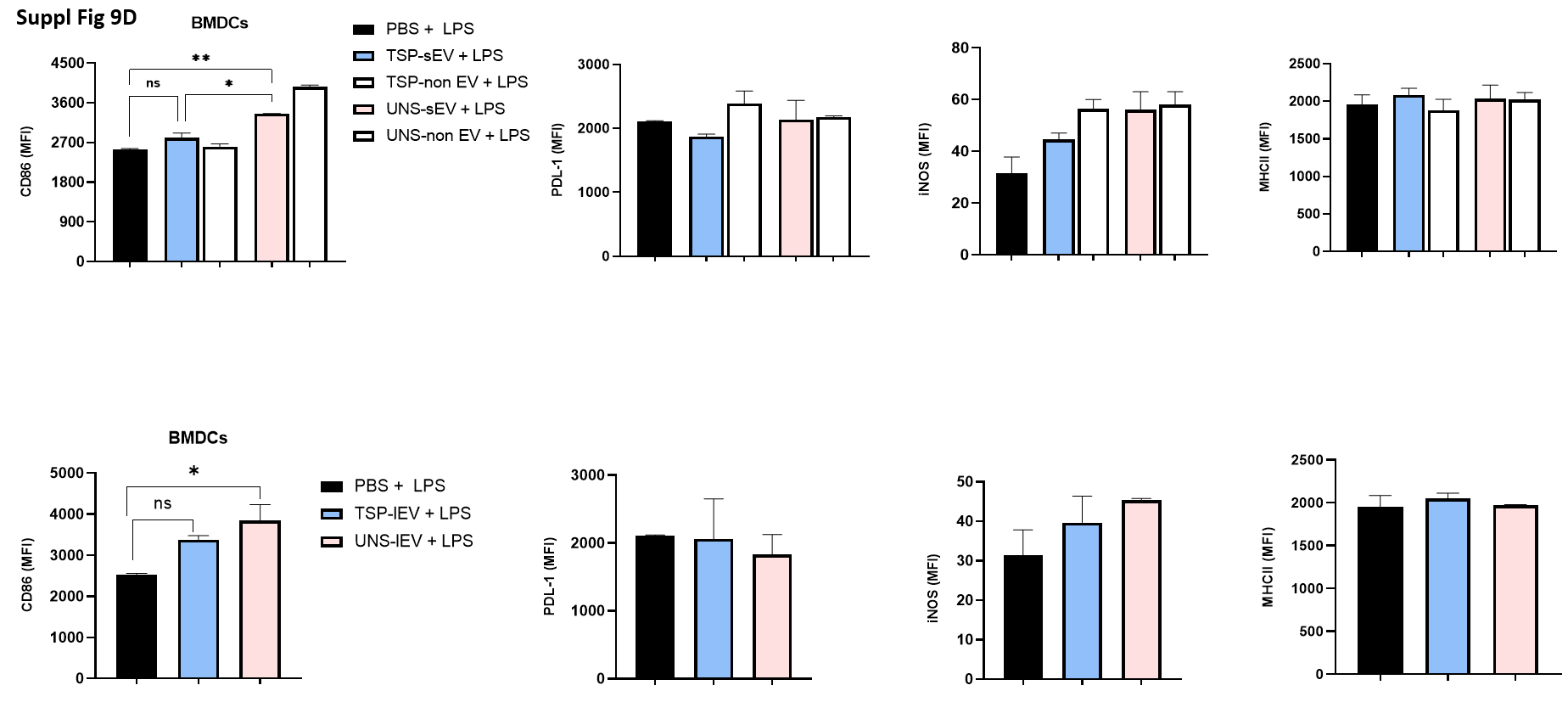


**Suppl Fig 9**. The effects of sEVs and lEVs on M1 markers and BMDCs in the presence or absence of LPS.

‎**(A)** CD86, MHCII, and iNOS expression were measured in BMDMs treated with PBS (control), TSP-sEVs, or UNS-sEVs in the presence or absence of LPS for 24h. UNS-sEVs ‎increased CD86 and MHCII in both naive and LPS-treated BMDMs relative to PBS and TSP-sEVs groups. Expression of iNOS as another M1 marker was not affected ‎with sEVs. Results were normalized and presented relative to PBS control. **(B)** CD86, MHCII, and iNOS expression were measured in BMDMs treated with PBS (control), TSP-lEVs, or UNS-lEVs in the presence or absence of LPS for 24h. UNS-lEVs increased CD86 and MHCII in both naive and LPS-treated BMDMs relative to PBS and TSP-lEVs groups. Expression of iNOS was significantly suppressed with TSP-lEVs in the presence of LPS. MHCII was also slightly downregulated by TSP-lEVs relative to PBS in the absence of LPS. **(C and D)** CD86, PDL-1, MHCII, and iNOS expression were measured in BMDCs treated with PBS (control), TSP-sEVs, or UNS-sEVs in the presence or absence of LPS for 24h. Data were presented as mean ± SEM of two independent experiments.


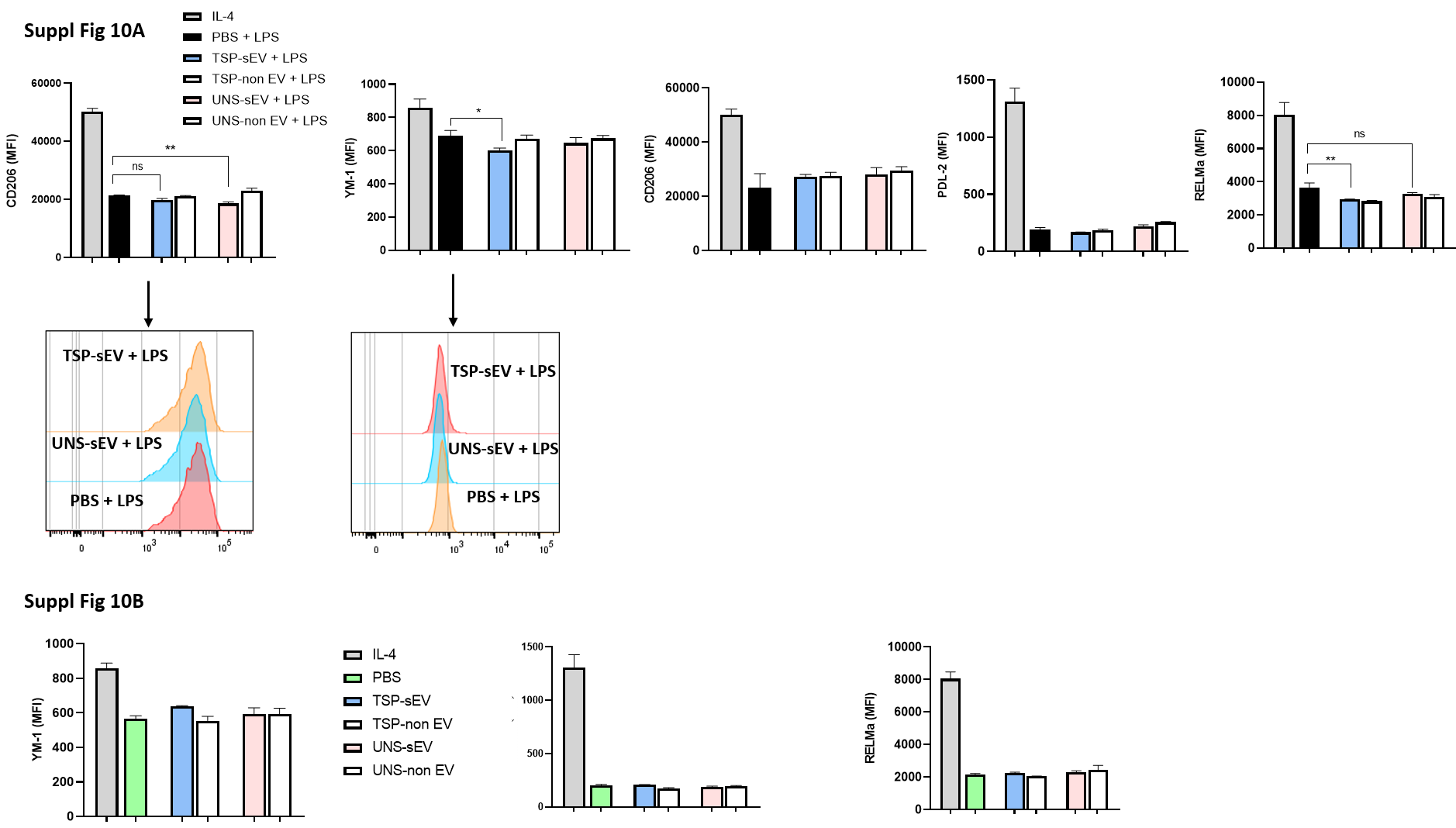


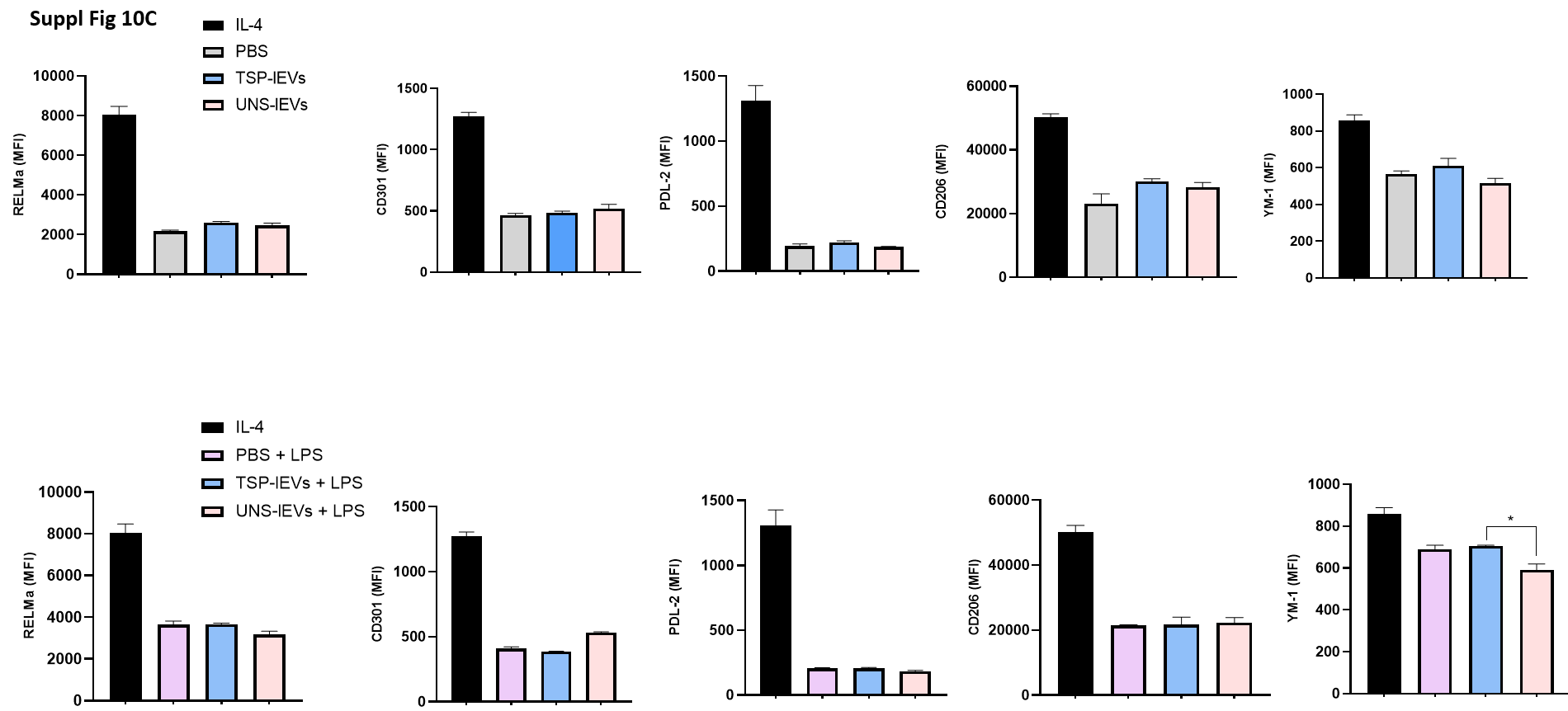


**Suppl Fig 10**. TSP-sEVs slightly downregulate RELMα and YM-1 markers in comparison with PBS group in the presence of LPS.

**(A)** BMDMs were stimulated with TSP-sEVs or UNS-sEVs with or without LPS for 24h and expression level of M2 markers was determined by flow cytometry. PBS and IL-4 were included as controls. The expression of YM-1 and RELMα were downregulated by TSP-sEVs relative to PBS in the presence of LPS. **(B)** However, no difference was observed in the absence of LPS. Expression of other M2 markers including CD206 and PDL-2 was not affected by sEVs. **(C)** None of M2 markers were significantly affected by lEVs. BMDMs were stimulated with TSP-lEVs or UNS-lEVs with or without LPS for 24h. PBS and IL-4 were included as controls. The expression of YM-1 and RELMα were downregulated by TSP-lEVs relative to PBS in the presence of LPS. However, no difference was observed in the absence of LPS. Data were presented as mean ± SEM of two independent experiments.


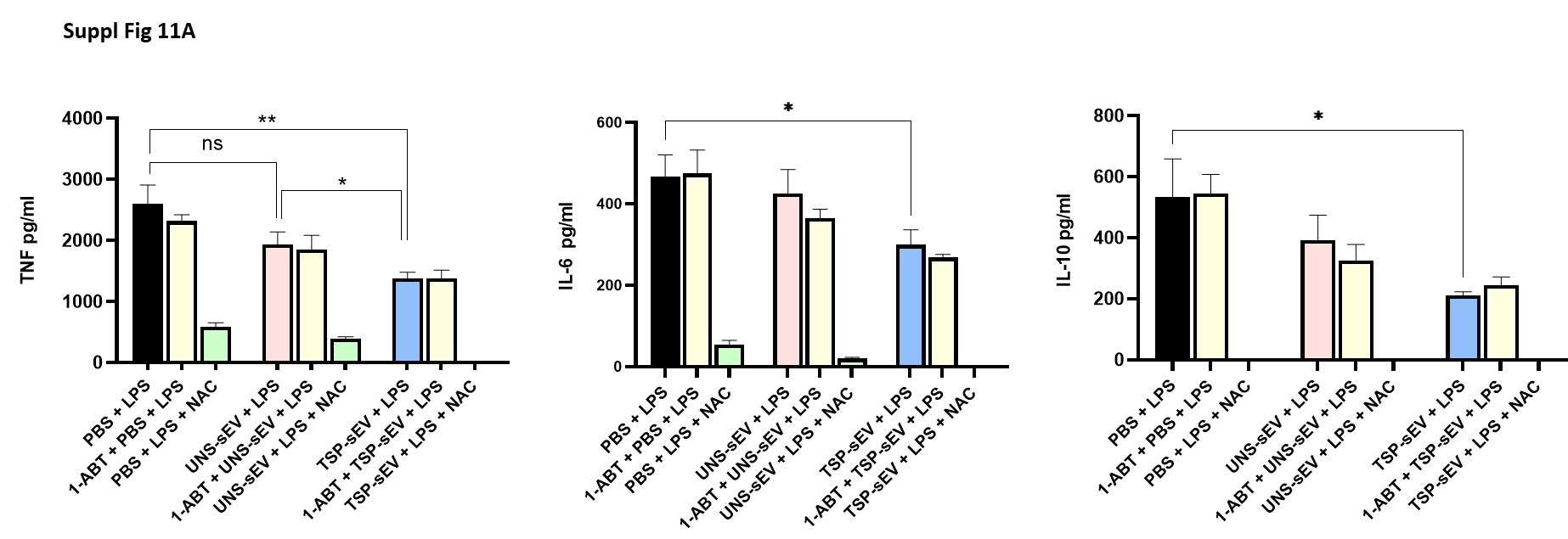


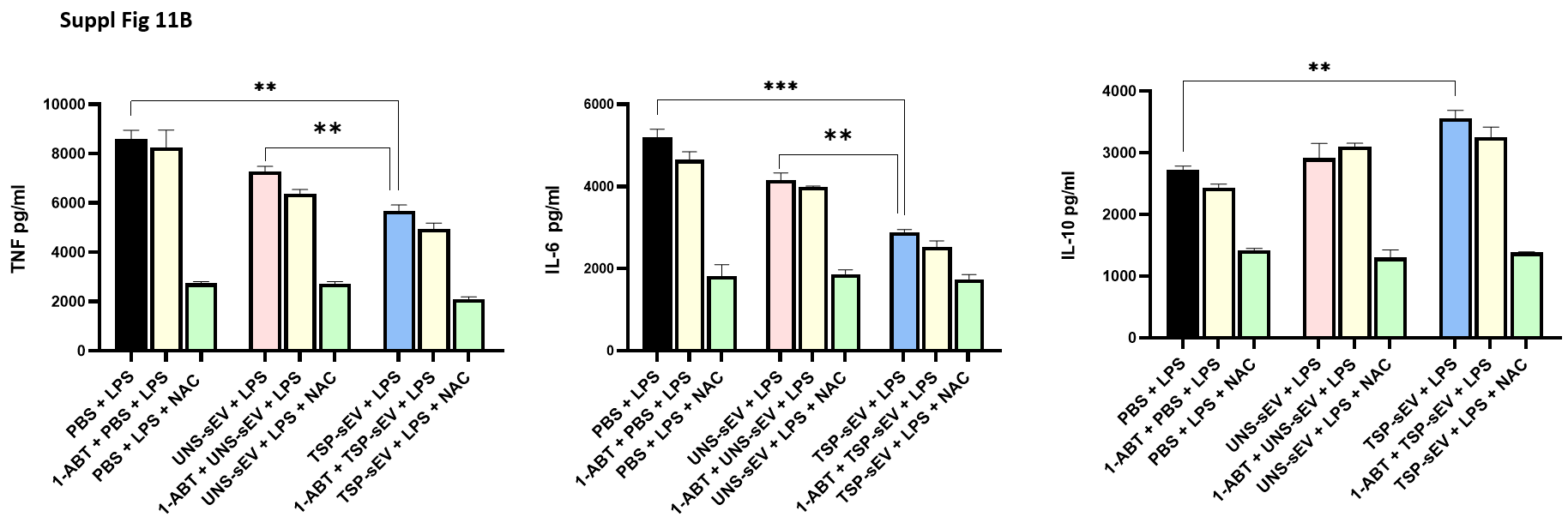


**Suppl Fig 11**. Stimulation of BMDMs with 1-ABT at 1mM has no effect on cell functionality.

**(A)** Pre-treatment of BMDMs with 1-ABT (1mM) for 0.5h before stimulation with EVs ‎showed no effect on BMDMs functionality in terms of cytokine after 6h and **(B)** 24h. In addition, **(C)** dose-response experiment on BMDMs exposed to 1-ABT showed that ‎‎1mM of 1-ABT is the lowest concentration which does not affect ROS generation in the ‎BMDMs.‎


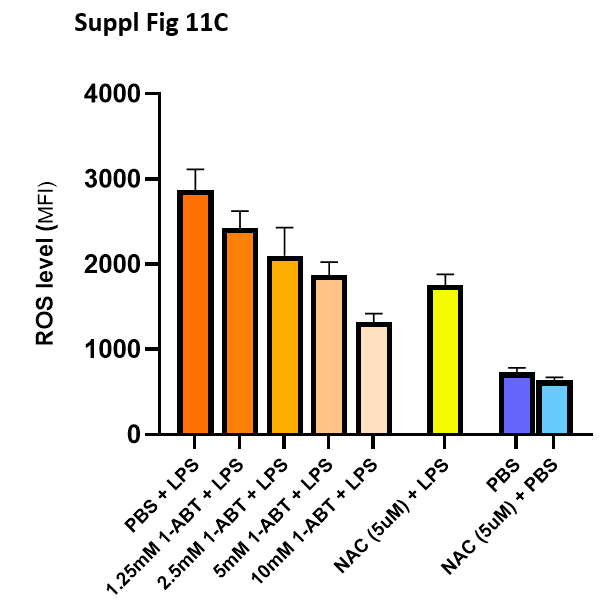


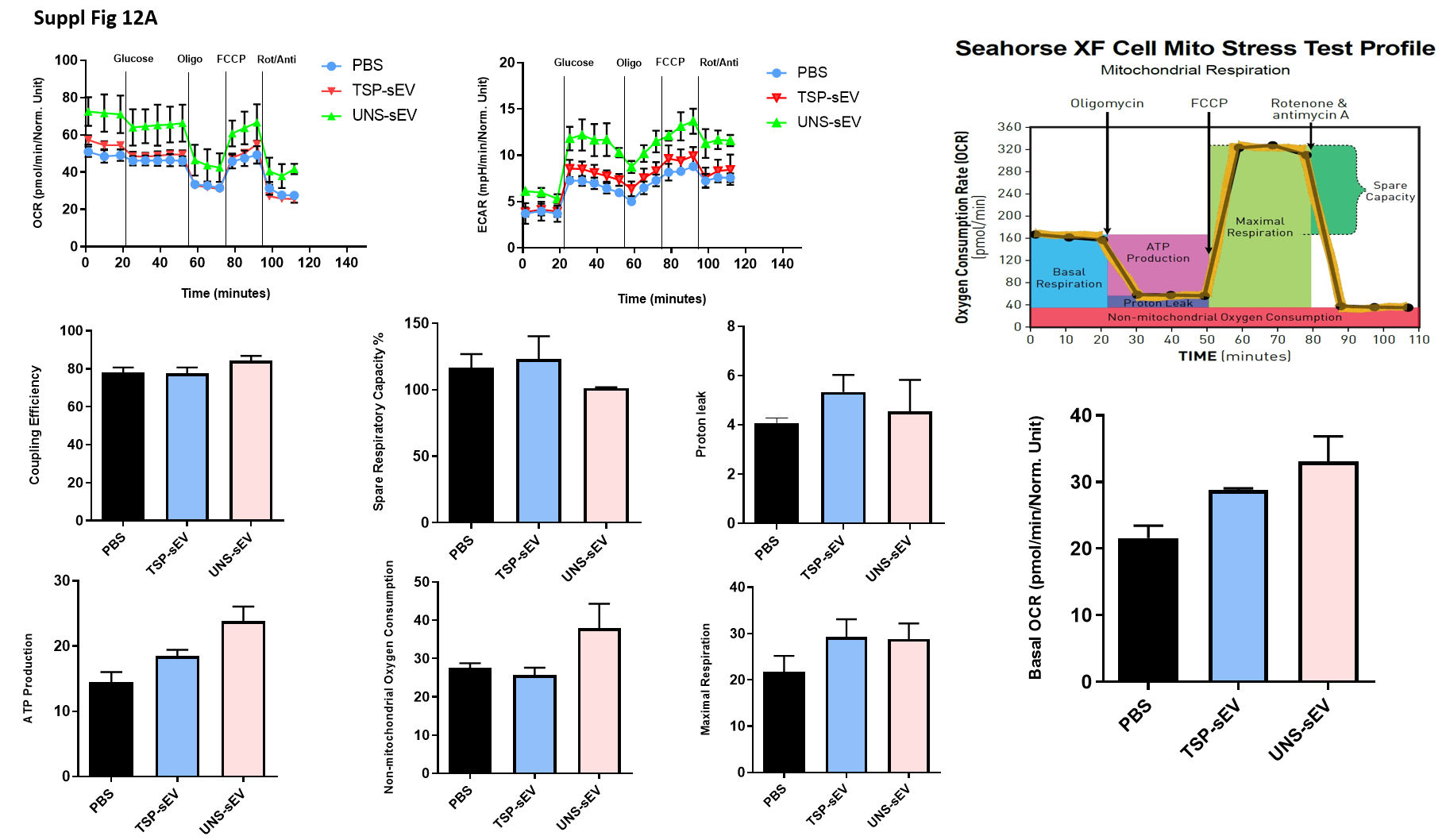


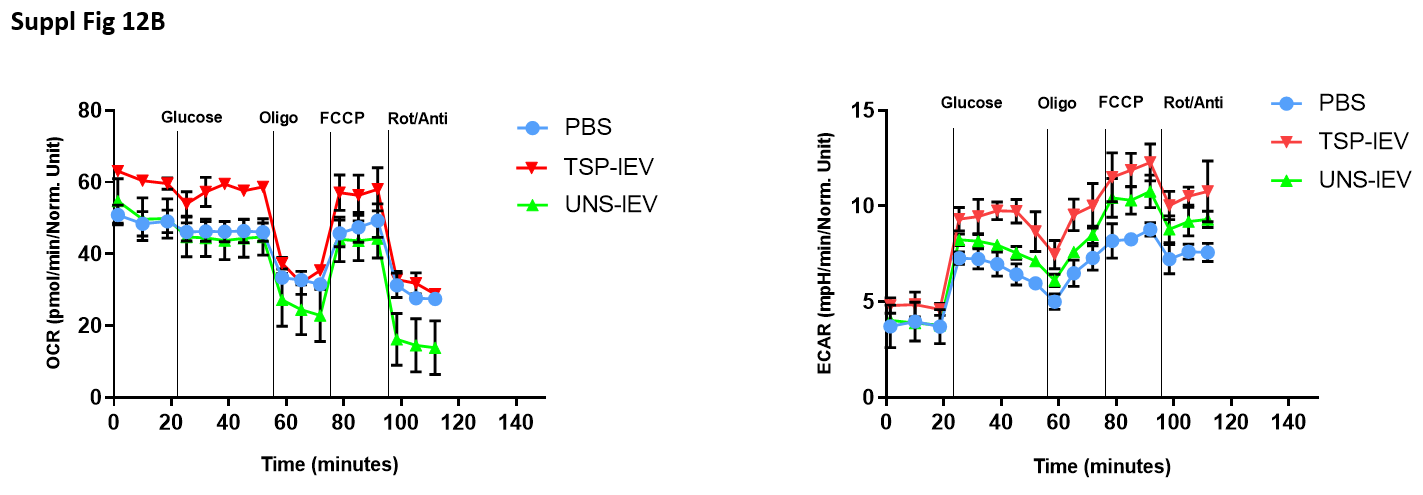


**Suppl Fig 12**. Effect of sEVs and lEVs on mitochondrial respiration and glycolysis in recipient cells.

**‎(A)** Graph of OCR and ECAR level after glucose, oligomycin, FCCP, and rotenone/antimycin treatment of macrophages incubated with sEVs derived from TSP-pulsed ‎or unstimulated BMDMs for 24h. Bar graph represents the average of basal OCR calculated from graph A. Other Bar graphs represent the average of Spare ‎Respiratory Capacity, ATP production, protein leak, and coupling efficiency calculated from graph A. No significant difference was observed between groups.‎ **(B)** Graph of OCR and ECAR level after glucose, oligomycin, FCCP, and rotenone/antimycin treatment of macrophages incubated with lEVs derived from TSP-pulsed ‎or unstimulated BMDMs for 24h. Data represent the mean ±SEM of 2 independent experiments, *p < 0.05 vs PBS.‎

**Suppl table 1. List of shared proteins between TSPs and TSP-sEVs**

|  | |  |  |  |  |
| --- | --- | --- | --- | --- | --- |
| **Protein ID** | **Gene** | **Protein** |  | **Organism** |  |
| B1AZS9 | **Prdx4** | **Peroxiredoxin-4** |  | *Mus musculus (Mouse)* | |
| P07724 | **Alb** | **Albumin** |  | *Mus musculus (Mouse)* | |
| P0DP28 | **Calm3** | **Calmodulin-3** |  | *Mus musculus (Mouse)* | |
| Q5SW88 | **Rab1a** | **RAB1A, member RAS oncogene family** | | *Mus musculus (Mouse)* | |
| Q792Z1 | **Try10** | **Trypsin 10** |  | *Mus musculus (Mouse)* | |
| Q9CPN9 | **2210010C04Rik** | **RIKEN cDNA 2210010C04 gene** | | *Mus musculus (Mouse)* | |
| P38647 | **Hspa9** | **Stress-70 protein, mitochondrial** | | *Mus musculus (Mouse)* | |
| A0A494BAN1 | **Ube2d2a** | **Ubiquitin-conjugating enzyme E2D 2A** | | *Mus musculus (Mouse)* | |
| P62264 | **Rps14** | **40S ribosomal protein S14** |  | *Mus musculus (Mouse)* | |
| E9Q0W8 | **Snrpe** | **Small nuclear ribonucleoprotein E** | | *Mus musculus (Mouse)* | |
| P62320 | **Snrpd3** | **Small nuclear ribonucleoprotein Sm D3** | | *Mus musculus (Mouse)* | |
| P63038 | **Hspd1** | **60 kDa heat shock protein, mitochondrial** | | *Mus musculus (Mouse)* | |
| P68372 | **Tubb4b** | **Tubulin beta-4B chain** |  | *Mus musculus (Mouse)* | |
| P68510 | **Ywhah** | **14-3-3 protein eta** |  | *Mus musculus (Mouse)* | |
| Q01853 | **Vcp** | **Transitional endoplasmic reticulum ATPase** | | *Mus musculus (Mouse)* | |
| Q02053 | **Uba1** | **Ubiquitin-like modifier-activating enzyme 1** | | *Mus musculus (Mouse)* | |
| Q68FD5 | **Cltc** | **Clathrin heavy chain 1** |  | *Mus musculus (Mouse)* | |
| Q9CWF2 | **Tubb2b** | **Tubulin beta-2B chain** |  | *Mus musculus (Mouse)* | |
| Q8BTZ7 | **Gmppb** | **Mannose-1-phosphate guanyltransferase beta** | | *Mus musculus (Mouse)* | |
| Q91ZJ5 | **Ugp2** | **UTP--glucose-1-phosphate uridylyltransferase** | | *Mus musculus (Mouse)* | |
| Q922F4 | **Tubb6** | **Tubulin beta-6 chain** |  | *Mus musculus (Mouse)* | |
| Q9CQV8 | **Ywhab** | **14-3-3 protein beta/alpha** |  | *Mus musculus (Mouse)* | |
| Q9QXK3 | **Copg2** | **Coatomer subunit gamma-2** |  | *Mus musculus (Mouse)* | |
| A0A085LRF5 | **M513_11595** | **Glucose-6-phosphate 1-dehydrogenase** | | *Trichuris suis (pig whipworm)* | |
| A0A085LRJ6 | **M513_11511** | **26S proteasome regulatory subunit 7** | | *Trichuris suis (pig whipworm)* | |
| A0A085M120 | **D918_08898** | **40S ribosomal protein S4** |  | *Trichuris suis (pig whipworm)* | |
| A0A085MDH4 | **M513_03911** | **Uncharacterized protein** |  | *Trichuris suis (pig whipworm)* | |
| A0A085MFU8 | **M513_02872** | **Malate dehydrogenase** |  | *Trichuris suis (pig whipworm)* | |
| A0A085MHX7 | **M513_02500** | **Uncharacterized protein** |  | *Trichuris suis (pig whipworm)* | |
| A0A085MKP3 | **M513_01459** | **Uncharacterized protein** |  | *Trichuris suis (pig whipworm)* | |
| A0A085NMH2 | **D918_09484** | **Peptidyl-prolyl cis-trans isomerase** | | *Trichuris suis (pig whipworm)* | |
| A0A085NTN8 | **M513_12817** | **Tr-type G domain-containing protein** | | *Trichuris suis (pig whipworm)* | |
| A0A085M2H0 | **M513_07629** | **Uncharacterized protein** |  | *Trichuris suis (pig whipworm)* | |
